# Permanently Charged Cationic Lipids—Evolution from Excipients to Therapeutic Lipids

**DOI:** 10.1002/smsc.202300270

**Published:** 2024-06-11

**Authors:** Pushpa Ragini S, Rajkumar Banerjee, Calum J. Drummond, Charlotte E. Conn

**Affiliations:** ^1^ Academy of Scientific and Innovation Research (AcSIR) Ghaziabad 201002 India; ^2^ Department of Oils, Lipid Science and Technology CSIR‐Indian Institute of Chemical Technology Hyderabad 500 007 India; ^3^ School of Science STEM College RMIT University 124 La Trobe Street Melbourne VIC 3000 Australia

**Keywords:** fusogenicity, molecular hybridization, permanently charged cationic lipids, serum compatibility, targeting

## Abstract

Cationic lipids are crucial in medical and biotechnological applications including cellular transfection and gene delivery. Ionizable cationic lipids are critical components of the mRNA‐based COVID vaccines while permanently charged cationic lipids have shown promise in cancer treatment. Despite significant research progress over the past few decades in designing improved, biocompatible cationic lipids, their transfection efficiency remains lower than that of viral vectors. Cationic lipids with additional functionalities like fusogenicity, stimuli‐responsiveness, targeting capabilities, and therapeutic activity have been engineered to improve their performance. This review highlights the importance of molecular hybridization toward the design of biocompatible cationic lipids having fusogenic, stimuli‐responsive, targeting, or therapeutic properties. This review mainly focuses on cationic lipids, having a permanent positive charge in the headgroup region, as these are typically employed to both increase cellular interactions and for improved loading, particularly for anionic nucleic acid‐based therapeutics and vaccines. Structure–activity relationships between the lipid chemical structure (headgroup, spacer, hydrocarbon chain) and, to a lesser extent, the self‐assembled nanostructure and the intrinsic biological activity of the multi‐functional cationic lipids are described. Finally, the challenges involved in developing smart lipids without affecting their inherent capacity to self‐assemble into structured nano‐carriers are discussed.

## Graphical abstract

In this review, the development of smart cationic lipids, typically formed via molecular hybridization of a lipid with a known bioactive moiety, is summarized. These multifunctional lipids may be considered as functional excipients, which may enhance the biological activity of certain drugs, and/or possess additional functionality such as fusogenic, targeting, or stimuli‐responsive properties.

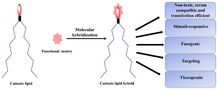

## 1 Introduction

Complex diseases such as cancer and microbial infections are multifactorial in nature with numerous redundant signalling pathways involved in the disease development.^[^ ^1^, ^2^, ^3^ ^]^ Despite recent advances in drug development, the complex pathophysiology of such diseases can result in drug resistance leading to a reduction in the treatment efficacy.^[^ ^4^, ^5^, ^6^, ^7^, ^8^ ^]^ This is particularly true for conventional monotherapy due to the inability of a single drug to manipulate the multiple targets associated with the pathogenesis of many diseases. Hence, complex diseases warrant the use of polytherapy or combination therapy in which two or more drugs are administered which act synergistically to target the multiple pathways responsible for the disease development. The use of combination therapy has improved clinical outcomes for a range of complex diseases including hypertension,^[^ ^9^, ^10^ ^]^ diabetes,^[^ ^11^, ^12^ ^]^ microbial infections,^[^ ^13^, ^14^ ^]^ neurodegenerative diseases,^[^ ^15^, ^16^ ^]^ and cancer.^[^ ^17^, ^18^, ^19^ ^]^

Drug nanocarriers can co‐encapsulate combinations of multiple drugs within a single nanocarrier, thereby successfully manipulating the pharmacokinetics of the drugs and potentially allowing for the simultaneous delivery of all drugs to the site of action.^[^ ^20^, ^21^ ^]^ Nanocarriers are typically preferred for combinations which include fragile biomolecules such as protein drugs or siRNA, due to issues including enzymatic degradation en route to the target site, rapid clearance, low aqueous solubility, and poor cellular uptake due to their generally larger size and/or charge. Nanocarriers can protect the encapsulated drugs from chemical or enzymatic degradation in vivo and, depending on the nanoparticle size, may also prevent renal clearance. This can reduce the required drug dosage with a concomitant reduction in adverse side‐effects. Drug nanocarriers fall into two broad categories: soft colloidal nanocarriers (typically lipid‐ or polymer‐based)^[^ ^22^ ^]^ and hard inorganic nanocarriers.^[^ ^23^, ^24^ ^]^ Hard inorganic nanoparticles, including quantum dots,^[^ ^25^ ^]^ gold nanoparticles,^[^ ^26^, ^27^, ^28^ ^]^ mesoporous silica nanoparticles,^[^ ^29^, ^30^, ^31^ ^]^ and superparamagnetic iron oxide nanoparticles (SPIONs),^[^ ^32^ ^]^ are often associated with high toxicity and low drug entrapment efficiency.^[^ ^33^ ^]^

Within the range of soft colloidal nanocarriers available, cationic nanocarriers having a positively charged surface are often preferred due to their preferential interaction with the anionic cell membrane, resulting in improved cellular uptake.^[^ ^34^ ^]^ For anionic bioactives, including nucleic acid‐based therapies and vaccines such as siRNA and mRNA, this has the additional advantage of improving the drug loading capacity. While both lipid‐ and polymer‐based cationic nanocarriers have been developed, cationic lipids may be preferred due to their ease of production and typically high biocompatibility.^[^ ^35^ ^]^ However, toxicity issues have been reported for cationic lipids both in vitro and in vivo.^[^ ^36^, ^37^ ^]^ There is, therefore, an urgent need for the development of cationic lipids with reduced toxicity. In addition to achieving high therapeutic efficacy of the encapsulated drugs, the design of cationic lipid‐based nanocarriers with targeted and controlled‐release capability is required. Employing molecular hybridization as a tool, many researchers are working toward the development of smart cationic lipids with lower toxicity, and containing added functionalities such as targeting, to improve therapeutic outcomes in complex diseases such as cancer and microbial infections.

In this review, we focus on the development of smart cationic lipids which may be formulated into lipid‐based nanocarriers. Lipids are amphiphilic organic molecules containing a non‐polar (hydrophobic) hydrocarbon chain region and a polar (hydrophilic) head group and may be considered as the excipients of lipid‐based drug delivery systems.^[^ ^38^ ^]^ Based on the charge they bear, lipids are classified as anionic (negatively charged – either ionizable or permanently charged), cationic (positively charged—either ionizable or permanently charged), and neutral (zwitterionic or nonionic). Upon addition of water, the hydrophobic effect drives the self‐assembly of lipids into a range of different morphologies, which is dictated by the shape of the lipid, frequently described by a critical packing parameter (CPP). The CPP is given as CPP = *v*_o_/*al*_o_ where *v*_o_ and *l*_o_ are the effective volume and the length of the surfactant tail and a is the effective surface area of the headgroup of the lipid.^[^ ^39^ ^]^ Based on the CPP value, lipids can self‐assemble into a wide range of nanoparticles; cylindrical‐shaped lipids with CPP ≈1 self‐assemble into the bilayer‐based lamellar phase, cone‐shaped lipids having a large head group and low hydrophobic volume (CPP < 1) generally form micelles, while wedge‐shaped lipids with CPP > 1 adopt inverse phases including the inverse bicontinuous cubic phases, the inverse hexagonal phase and inverse micelles. We note that in order to achieve many of the different self‐assembly objects the hydrophobic lipid chains need to be in a molten (fluid) state.

**Figure 1:**
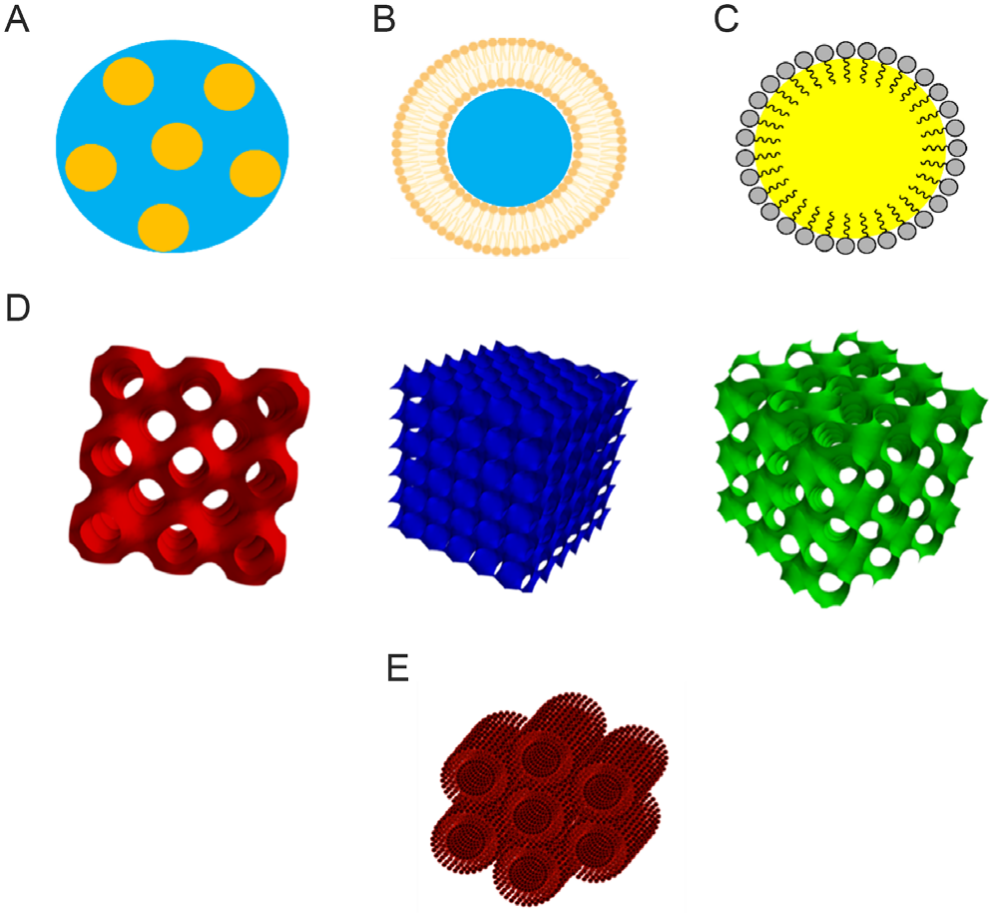
Lipid nanoparticles A) Emulsions (droplets of oil in water) B) Unilamellar liposome (aqueous layer surrounded by a lipid bilayer) C) Solid lipid nanoparticle (SLNs) (spherical particles with solid lipid matrix and a surfactant layer to stabilize the SLNs in aqueous phase) D) Cubosomes (bicontinuous lipid membrane with two non‐intersecting water channels) E) Hexosomes (seven subunits packed into a hexagonal arrangement). (D) Reproduced with permission.^[^ ^144^ ^]^ Copyright 2021 Elsevier Inc. and (E) Reproduced with permission.^[^ ^49^ ^]^ Copyright 2020, Elsevier Inc.

Lipid‐based nano‐formulations may be classified as emulsions (**Figure** **1**A),^[^ ^40^ ^]^ vesicular systems (Figure 1B),^[^ ^41^ ^]^ and SLNs (Figure 1C).^[^ ^42^ ^]^ Recent research effort has also focussed on the development of more complex lyotropic liquid crystalline bulk phases^[^ ^43^ ^]^ including the inverse bicontinuous cubic (Q_II_) phases (Figure 1D) and hexagonal (H_II_) phases (Figure 1E) and their corresponding nano‐ or sub‐micron particles (cubosomes and hexosomes).

To date, research on the use of lipid‐based nanocarriers for drug delivery has focussed on emulsions, liposomes, or solid lipid nanoparticles (SLNs), which may be formulated from either natural or synthetic lipids, with typical size ranging from 10 to 400 nm. Lipid‐based emulsions, Figure 1A, include microemulsions, nano emulsions, self‐micro emulsifying drug delivery systems, and self‐nanoemulsifying drug delivery systems.^[^ ^44^ ^]^ Liposomes,^[^ ^45^ ^]^ niosomes,^[^ ^46^ ^]^ and virosomes^[^ ^47^, ^48^ ^]^ are types of vesicular nanocarriers. In liposomes, Figure 1B, lipid molecules form a closed bilayer shell (suitable for encapsulation of lipophilic drugs) enclosing an aqueous interior which may be used to encapsulate water‐soluble drugs.^[^ ^45^ ^]^ Niosomes are vesicular delivery systems formed by the self‐assembly of non‐ionic surfactants,^[^ ^46^ ^]^ while virosomes consist of liposomes containing viral envelope proteins. SLNs, Figure 1C, are generally spherical nanoparticles containing a lipophilic core that is solid at room temperature. Of late, nanoparticles of more complex symmetry including cubosomes or hexosomes, have been developed as drug delivery vehicles. The advantages of cubosomes (Figure 1D), and hexosomes (Figure 1E) can include high biocompatibility,^[^ ^49^ ^]^ high drug loading capacity and partially controllable size, and morphology enabling encapsulation of drugs of a range of different molecular weights.^[^ ^50^, ^51^, ^52^ ^]^

To date, the clinical application of lipid nanocarriers for drug delivery has mainly focussed on liposomes. Some liposomal delivery systems include Doxil for the delivery of doxorubicin, used to treat certain kinds of ovarian cancer,^[^ ^53^ ^]^ AmBiSome to deliver amphotericin B for the treatment of fungal infections^[^ ^54^ ^]^ and Depodur, an extended release liposome injection of morphine sulphate for the treatment of post‐operative pain.^[^ ^55^ ^]^ One of the first examples of a lipid nanocarrier was CPX‐351 (Vyxeos), a liposomal system for the co‐delivery of daunorubicin and cytarabine, which increased the median overall survival for patients with acute myeloid leukemia (AML); FDA approval was gained for clinical use in 2017.^[^ ^56^ ^]^ Vyxeos liposomes are made of mixture of phospholipids (DSPC, DSPG) and cholesterol in the ratio 7:2:1. While the recent development of the Pfizer BioNTech mRNA‐based COVID vaccines has exemplified the global use of lipid nanoparticles containing an ionizable lipid,^[^ ^57^, ^58^ ^]^ we note that, to date, there has been no successful clinical translation of a lipid nanocarrier containing a permanently charged cationic lipid, potentially due to toxicity issues arising from either alterations to the biosynthesis and metabolism of cholesterol, steroid and lipids, and the induction of reactive oxygen species (ROS) or proinflammatory activity. There is therefore a pressing need for an improved understanding of the structure–property relationships governing the efficacy and toxicity of cationic lipids to allow for the design of a new generation of cationic lipids with enhanced functionality and lowered toxicity.

## 2 Evolution of Commercially Available Cationic Lipids

Initial research interest in the development and use of cationic lipids in medical applications was driven by their application as non‐viral transfection agents for nucleic acid‐based therapies. Issues with the clinical translation of viral vectors due to their inherent immunogenicity, mutagenesis, limitations on the size of encapsulated DNA, and production difficulties^[^ ^59^ ^]^ led to their increasing replacement by synthetic cationic lipids which were easier to manufacture and demonstrated an improved safety profile (i.e., were non‐immunogenic and non‐oncogenic).^[^ ^60^ ^]^ Unlike viral vectors which rely on biological interactions to pack nucleic – acid‐based molecules, cationic lipids can encapsulate nucleic acids via electrostatic interactions. The pioneering work by Felgner demonstrated, for the first time, that the cationic lipid, DOTMA (*N*‐[1‐(2,3‐dioleyloxy) propyl]‐*N, N,N*‐trimethylammonium chloride), a quaternary ammonium salt, successfully transfected DNA in mammalian cells.^[^ ^61^ ^]^ The excess positive charge in the headgroup of these lipids could facilitate adhesion to the cell membrane. The commercially available liposomal gene transfer vector Lipofectin consists of a combination of the cationic lipid DOTMA with the fusogenic neutral lipid DOPE (dioleoylphosphatidylethanolamine).^[^ ^62^ ^]^ Following DOTMA, many other related cationic lipids were developed via minor chemical modifications to the basic architecture; these are summarised in **Table** **1**. For example, the cationic lipid DOTAP (1,2‐dioleoyloxy‐3‐(trimethylammoniopropane chloride) was designed by replacing the diether groups with biodegradable diester bonds; the resulting increase in biodegradability meant that DOTAP was observed to accumulate less in tissues. Later, other synthetic cationic lipids were generated via modifications to either the quaternary ammonium group in the headgroup region or the lipid chain; substitution with a 14 carbon saturated alkyl chain resulted in DMRIE (1,2‐dimyristyloxypropyl‐3‐dimethyl‐hydroxyethyl ammonium bromide) while substitution with a 12 carbon saturated alkyl chain in the hydrophobic region and replacing a methyl group with the propylene amine in the head region resulted in DLRIE ((+/−)–*N*‐(3‐aminopropyl)‐*N,N*‐dimethyl‐2,3‐*bis*(dodecyloxy)‐1‐propanaminium bromide).^[^ ^63^ ^]^ In vivo studies demonstrated that GAP‐DLRIE:DOPE liposomes exhibited 15 times higher transfection efficiency inside arteries compared to either the naked reporter gene, or transfection of the gene with DMRIE:DOPE liposomes.^[^ ^64^ ^]^ DODAB (dimethyldioctadecylammonium bromide) consists of a hydrophobic domain bearing twin 18 carbon chains directly attached to the quaternary ammonium head group. The positive charge of DOSPA (2,3‐dioleyloxy‐*N*‐[2‐(sperminecarboxamido) ethyl]‐*N,N*‐dimethyl‐1‐propanaminium chloride) was increased by conjugating spermine to the head group via an ethylene spacer.

**Table 1:** Chemical structure of selected commercial cationic lipids. Adapted with permission.^[^ ^146^ ^]^ Copyright 2020, American Chemical Society.

Commercial cationic lipids	Structure
DOTMA	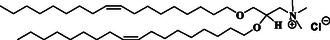
DOTAP	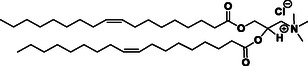
DMRIE	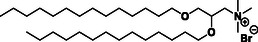
GAP‐DLRIE	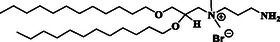
DODAB	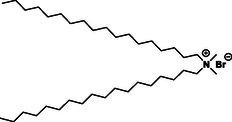
DOSPA	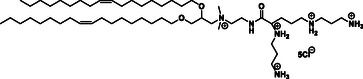

Although cationic lipids are widely employed for laboratory transfection purposes owing to their improved safety profiles compared to viral vectors, their reported high toxicity has hindered their successful clinical translation. Cationic lipids have been demonstrated to cause cytotoxicity by altering the biosynthesis and metabolism of cholesterol, lipids, and steroids,^[^ ^65^ ^]^ interaction with enzymes such as protein kinase C,^[^ ^66^ ^]^ membrane destabilization, and oxidative stress,^[^ ^67^ ^]^ hemolysis by rupturing the fragile red blood cell membrane,^[^ ^37^ ^]^ immunogenicity via interactions with a variety of proteins (extracellular or intracellular),^[^ ^36^ ^]^ and rapid plasma clearance due to opsonization.^[^ ^68^ ^]^ Their toxicity is mainly attributed to their positively charged head group, although other molecular moieties may also contribute. Cationic lipid particles are reported to undergo aggregation and accumulation in the lung, liver, and spleen resulting in toxicity, especially lipids bearing an ether linkage which are less prone to biodegradation.^[^ ^38^, ^69^ ^]^ Lipoplexes made of commercial cationic lipids like DMRIE, DOTMA, and DOTAP are known to trigger inflammatory reactions upon systemic administration.^[^ ^69^ ^]^ Research attention has, therefore, been directed to the rational design of less toxic lipids, via structural modifications in the basic lipid architecture.

**Figure 2:**
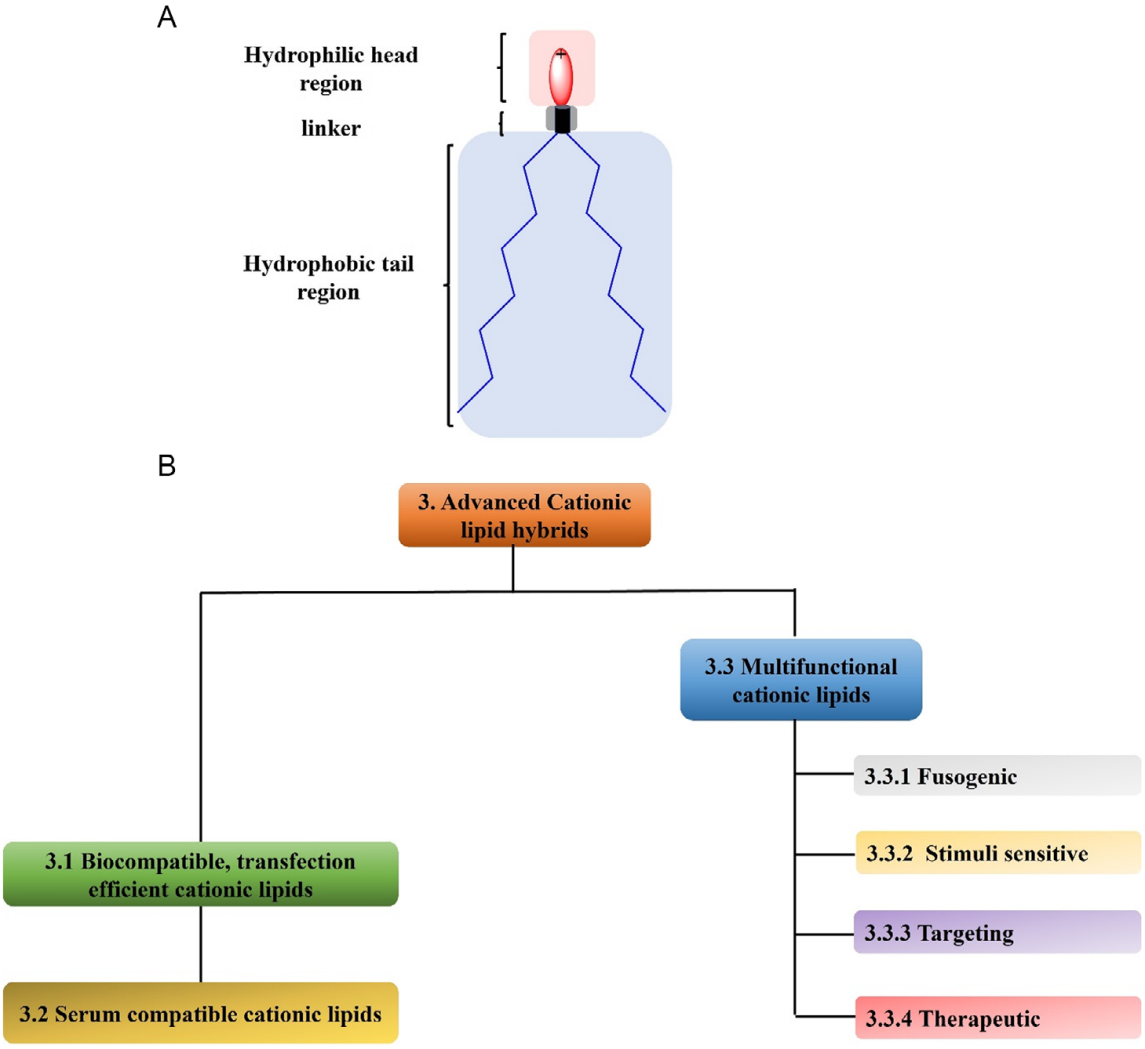
A) Basic architecture of the lipid. B) Classification of advanced cationic lipids into 3.1 biocompatible and improved transfection efficiency cationic lipids, 3.2 serum compatible cationic lipids and 3.3 cationic lipids with additional functionalities 3.3.1 fusogenic 3.3.2 stimuli‐responsive 3.3.3 targeting and 3.3.4 therapeutic cationic lipids.

The relatively flexible basic lipid architecture, (**Figure** **2**A), consisting of a hydrophilic headgroup and a hydrophobic hydrocarbon chain region joined by a linker moiety, allows for a wide range of different chemical modifications to be carried out. Initial studies focussed on the reduction of toxicity were later extended to the synthesis of more advanced lipid hybrids capable of targeting specific cells, stimuli responsive drug release, endosomal escape properties, and enhanced therapeutic properties (Figure 2B). Herein, we critically review recent advances in the design and synthesis of smart cationic lipids containing rational chemical modifications to their molecular architecture which improve their transfection efficiency or drug delivery properties, while reducing any associated toxicity issues. They are capable of executing multiple actions and enhanced activity depending on the pharmacophores conjoined.^[^ ^70^, ^71^ ^]^

Due to the very large number of cationic lipids reviewed herein, in Section 3 we initially provide a general overview summarizing the main trends in cationic lipids with improved biocompatibility and transfection efficiency (Section 3.1), and serum stability (Section 3.2). In Section 3.3 we summarize recent developments in the synthesis of smart cationic lipids imparted with additional functionality such as fusogenic cationic lipids (Section 3.3.1), stimuli‐responsive cationic lipids (Section 3.3.2), targeted cationic lipids (Section 3.3.3), and therapeutic cationic lipids (Section 3.3.4). For a more in‐depth, comprehensive review of the field the reader is directed to Section 4 where additional information and detail are provided for each of these classes of cationic lipid. A discussion of the main conclusions and suggested future directions for research on cationic lipids is provided in Section 5. Note that, for each lipid described, we have retained the nomenclature used in the original published work.

## 3 Advances in Cationic Lipid Design: General Overview

A range of studies have looked at the effect of the chemical structure of the cationic lipid on its efficacy for a range of biomedical applications including transfection, targeting capability, and therapeutic functionality. The main trends observed in these studies are summarized in this section and **Table** **2**. Relatively few studies have assessed how the self‐assembly structure of the lipid, or their inclusion in a self‐assembly vehicle formed by another lipid, impacts their efficacy for the end‐use application.

**Table 2:** Summary of influence of different head groups, linkers and tails on the properties of cationic lipid hybrids.

	Head group	Linker/spacer	Hydrophobic region
Biocompatible cationic lipids with reduced toxicity	Inclusion of: • Heterocyclic rings: – Imidazolium ring^[^ ^72^ ^]^ – Pyridinium ring^[^ ^73^, ^74^ ^]^ • Guanidinium group^[^ ^75^ ^]^ • Natural molecules: – Lactic acid^[^ ^80^ ^]^ – Nucleobase uracil^[^ ^81^ ^]^	Inclusion of: • Ester group^[^ ^72^, ^73^, ^74^ ^]^ • Amide group^[^ ^74^ ^]^	Inclusion of naturally existing hydrophobic molecules: • Carotenoid^[^ ^78^ ^]^ • Cholesterol^[^ ^79^ ^]^
Cationic lipids with improved transfection efficiency	Inclusion of: • Polar groups in the head region – Guanidinium group, – Hydroxy group,^[^ ^72^, ^78^, ^79^, ^80^ ^]^ – Amine^[^ ^77^ ^]^ • Increasing charge to mass ratio (for gemini cationic lipids)^[^ ^74^, ^83^, ^136^ ^]^	Inclusion of: • Amide group^[^ ^139^ ^]^ Type of spacer (for gemini cationic lipids): – Short hydrophobic linker^[^ ^74^ ^]^ and – Oxyethylene spacer^[^ ^83^, ^136^ ^]^	Length of the saturated hydrophobic chains • C12^[^ ^80^ ^]^ • C14^[^ ^72^, ^74^ ^]^ • C18^[^ ^72^, ^81^ ^]^ Type and position of the unsaturation‐ triple bond, toward the end of the hydrophobic chain^[^ ^82^ ^]^ Shape of the molecule‐truncated cone shape^[^ ^74^ ^]^
Serum compatible cationic lipids	Dimeric head groups^[^ ^85^, ^86^, ^87^ ^]^ Polar moieties in the head group: • Hydroxy^[^ ^76^, ^78^, ^139^ ^]^ • Amine^[^ ^77^ ^]^	Spacer length: oxyethylene spacer (three unit length)^[^ ^84^ ^]^ Chemical nature of linker: *β* hydroxy‐triazole^[^ ^134^ ^]^	Cholesterol based gemini cationic lipids^[^ ^87^ ^]^
Fusogenic cationic lipids	Inclusion of: • quaternary ammonium group (imidazolium group)^[^ ^94^ ^]^	Inclusion of: • Amide groups^[^ ^90^ ^]^	• Unsaturation in the hydrocarbon chains^[^ ^91^, ^92^, ^93^ ^]^ • Trans unsaturation; position of the hydrocarbon chain (pyridinium based head group)^[^ ^91^ ^]^ • Asymmetry in the hydrocarbon chains^[^ ^93^ ^]^
Stimuli‐responsive cationic lipids	Inclusion of:	Inclusion of pH sensitive chemical groups: • Ester^[^ ^95^ ^]^ • Triazole ring^[^ ^139^ ^]^	Inclusion of: • Redox sensitive lipoic acid bearing reducible—S—S—bonds^[^ ^96^ ^]^
Targeting cationic lipids	Inclusion of: Ligands targeting specific receptors: • Haloperidol targeting sigma receptors^[^ ^105^ ^]^ • Folic acid targeting folate receptor,^[^ ^106^ ^]^ • Cyclic and open sugars targeting asialoglycoprotein receptors in the liver,^[^ ^110^ ^]^ • Shikimoyl, mannosyl, and quinoyl groups targeting mannose receptors in immune cells^[^ ^111^ ^]^ Ligands targeting specific transporters: • Nipecotic acid targeting the GABA transporter in the brain^[^ ^107^ ^]^ • Pyridinium ring targeting polyamine transporters in lung cells^[^ ^73^ ^]^ Ligands promoting non‐specific organ selective accumulation: • *β*‐Amphetamine with demonstrated ability to cross the blood–brain‐barrier^[^ ^109^ ^]^ • Cationic quaternary ammonium sulfonamide amino lipids with ability to accumulate in the lungs^[^ ^112^ ^]^	Spacer length‐dependent on the headgroup architecture with • C2 spacer best for open sugar headgroups • C6 spacer best for cyclic sugar headgroups^[^ ^110^ ^]^	Short saturated alkyl chain length • C8^[^ ^105^, ^106^ ^]^ Long saturated alkyl chain lengths • C16^[^ ^108^, ^109^, ^111^ ^]^ • C18^[^ ^112^ ^]^
Anticancer cationic lipids	Inclusion of: Steroid hormones • Estradiol^[^ ^71^ ^]^ • Progesterone^[^ ^117^ ^]^ • Dexamethasone^[^ ^118^ ^]^ • Hydrocortisone^[^ ^116^ ^]^ Pharmacophoric groups • Haloperidol^[^ ^105^ ^]^ • Stilbene Naturally occurring weakly active groups • Cordiarimide^[^ ^70^ ^]^ Molecules with negligible anti‐cancer activity • Benzamide^[^ ^114^, ^115^ ^]^	–	Anticancer activity was observed for cationic lipids with the following saturated hydrocarbon chain lengths: • Short: C8‐C10^[^ ^71^, ^105^, ^114^, ^117^, ^118^, ^121^ ^]^ • Medium:C12;^[^ ^70^ ^]^ • Long: C16^[^ ^116^, ^120^ ^]^
Antimicrobial cationic lipids	Inclusion of: Quaternary ammonium group into naturally existing weakly antimicrobial molecules^[^ ^131^, ^132^ ^]^ Bioactive head groups such as: • Oxazole^[^ ^133^ ^]^ • Benzamide^[^ ^134^ ^]^ Increase in the head group charge^[^ ^130^ ^]^	• 8 carbon spacer^[^ ^131^ ^]^ • 11 Carbon spacer^[^ ^131^, ^132^ ^]^	Antimicrobial activity was observed for cationic lipids with the following saturated hydrocarbon chain lengths: • Short: C7–C11^[^ ^131^, ^132^, ^133^, ^134^ ^]^ • Medium: C12^[^ ^132^ ^]^ • Long: C18^[^ ^131^ ^]^ The effect of chain length was dependent on the headgroup with the following saturated hydrocarbon chain lengths observed to be most active: • Sophorolipids: C18 and C11^[^ ^131^, ^132^ ^]^ • Lipoxazole: C8^[^ ^130^ ^]^ • Lipobenzamide: C9 and C11^[^ ^134^ ^]^ The ability to form micelles was associated with increased antimicrobial activity.^[^ ^131^, ^132^ ^]^
Antioxidant cationic lipids	Inclusion of: • Antioxidant phenolic moiety^[^ ^135^ ^]^		Longer chain lengths (e.g., saturated C15 chain) were shown to improve lipophilicity and insertion into the cell membrane^[^ ^135^ ^]^

### 3.1 Improved Biocompatibility and Transfection Efficiency

Although the first generation of cationic lipids were efficient for encapsulation of nucleic acids, their measured transfection efficiencies were often lower compared to viral vectors. In addition, the first‐generation of cationic lipids were associated with high toxicity, attributed to the highly dense localized positive charge of the head group. Hence, attempts were made to design cationic lipids with improved transfection efficiencies and biocompatibility.

**Figure 3:**
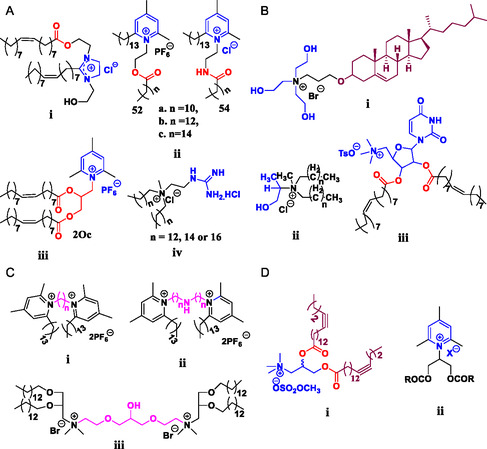
A) Cationic lipids bearing charge delocalizing heterocyclic rings i) imidazolium based DOTIM ii) pyridinium‐based 52b (ester), 54c (amide) iii) 2Oc, and iv) guanidinium head group B) Cationic lipids conjugated to naturally existing molecules i) cholesterol‐based Chol‐THEA ii) lactic acid‐based, lipid 4 (*n* = 12) and iii) nucleobase‐based DOTAU C) Gemini cationic lipids—pyridinium‐based i) hydrophobic linker (gemini surfactant 29 *n* = 2) and ii) hydrophilic linker and iii) cardiolipin‐based gemini cationic lipid (CCLA); spacer is indicated in pink D) i) dialkynoyl DOTAP congener (lipid analogue 4) and ii) truncate shaped cationic lipid with a pyridinium head group (lipid 9).

A variety of chemical modifications to the **headgroup region**, including the grafting of heterocyclic rings such as imidazolium (**Figure** **3**A(i)),^[^ ^72^ ^]^ pyridinium (Figure 3A(ii & iii)),^[^ ^73^, ^74^ ^]^ and electron‐rich guanidinium group,^[^ ^75^ ^]^ Table 2, was shown to both increase transfection efficiency and decrease toxicity. It is suggested that the reduced toxicity in this case reflects the charge delocalization, Table 2, over the heterocyclic ring or guanidinium group (Figure 3A(iv)). In addition, the presence of polar groups in a quaternary ammonium head group region (such as guanidinium groups,^[^ ^75^ ^]^ or hydroxy or amine groups in tocopherol, benzothiazole cationic lipid hybrids^[^ ^76^, ^77^ ^]^) generally increased the measured transfection efficiency, potentially due to increased hydrogen bonding between the cationic lipid and the nucleic acid molecule which can enhance uptake, as well as enhanced interactions between the cationic lipids and the typically anionic cell membrane resulting in increased cellular uptake. The conjugation of a naturally occurring molecule, such as a carotenoid molecule,^[^ ^78^ ^]^ cholesterol (Figure 3B(i)),^[^ ^79^ ^]^ lactic acid (Figure 3B(ii)),^[^ ^80^ ^]^ or uracil (Figure 3B(iii))^[^ ^81^ ^]^ was also shown to improve the biocompatibility and increase transfection efficiency.

Another effective strategy involves chemical modifications to the hydrocarbon chain region. The modification of the DOTAP chain to include a C–C triple bond toward the end of the chain (Figure 3D(i)) was shown to increase the rigidity of the lipoplex membrane forming a gel‐like structure with 2–3 times higher transfection efficiency.^[^ ^82^ ^]^

The charge per molecule, Table 2, can affect not only the transfection efficiency but also the cytotoxicity of the cationic lipids. Gemini cationic lipids (having two units of cationic charge per one molecule) based on pyridine (Figure 3C(i & ii))^[^ ^74^ ^]^ and cardiolipin (Figure 3C(iii))^[^ ^83^ ^]^ displayed higher transfection efficiencies and lower cytotoxicity compared to their monomeric counterparts. It has been suggested that such gemini lipids may self‐assemble at lower concentrations which could also increase transfection efficiency, although the effect of self‐assembly structure on transfection efficiency and toxicity remains poorly understood. For such gemini lipids, both the length of the hydrophobic chain and the length of the spacer were observed to influence the transfection efficiency, although this effect was cell‐line dependent. Gemini cationic lipids bearing penta methylene spacers have generally displayed the highest transfection efficiency to date. The shape of the lipid was also shown to play a role in improving the transfection efficiency. For example, a pyridinium cationic lipid (Figure 3D (ii)) with a truncated cone shape exhibited the highest transfection efficiency compared to the non‐truncated counterparts (**Figure A** **1**F).^[^ ^74^ ^]^

### 3.2 Serum Compatible Lipids

Serum compatibility is important to ensure the effective translation of lipid‐based transfection agents to in vivo studies. Commercially available formulations such as Lipofectamine and DOTAP/cholesterol have been shown to effectively transfect cells up to 80% added serum and this property must be assessed for formulations involving new cationic lipids.

**Figure 4:**
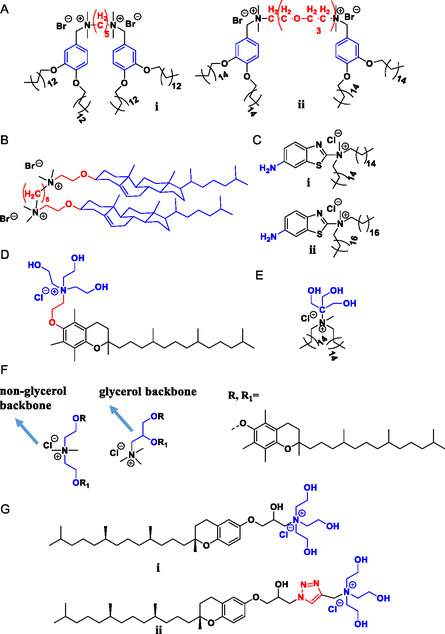
Cationic lipids with improved serum compatibility A) Gemini cationic lipids with aromatic backbone i) polymethylene spacer (lipid 5c) ii) oxyethylene spacer (lipid 4c) B) Cholesterol‐based gemini cationic lipid (lipid 2c) C) Amine bearing benzothiazole cationic lipid hybrids i) lipid 9 ii) lipid 10 D) Tocopherol‐based cationic lipids with oxyethylene spacer and hydroxyethyl head group, lipid 5 E) Tris cationic lipid hybrid, Tris lipid 1 F) Tocopherol cationic lipid hybrid with non‐glycerol (lipid 1) and glycerol backbone (lipid 2) G) tocopherol cationic lipid hybrids i) without *β* hydroxy‐triazole linker (Lp1) ii) With *β* hydroxy‐triazole linker (Lp 2).

Cationic lipids based on benzothiazole (**Figure** **4**C(i & ii)),^[^ ^77^ ^]^ tocopherol (Figure 4D,F,G),^[^ ^76^ ^]^ and Tris base (Figure 4E)^[^ ^84^ ^]^ exhibited serum compatibility with the transfection profiles unaffected by the presence of serum. The majority of studies which focussed on improved serum compatibility involved the introduction of polar groups, including hydroxy and amine groups, Table 2, to the cationic lipid headgroup. This was generally associated with increased serum compatibility by efficiently masking the positive charge of the cationic head group and reducing protein adsorption.

Some studies focussed on the development of gemini cationic lipids (based on an aromatic backbone (Figure 4A(i and ii)^[^ ^85^, ^86^ ^]^ and cholesterol (Figure 4B)^[^ ^87^ ^]^); the chemical identity of the spacer, the length of the spacer, and the length of the hydrocarbon chain were all shown to affect transfection efficiency in the presence of serum, Table 2, but no clear, universal trends were observed. Co‐formulation with the highly fusogenic lipid DOPE improved transfection efficiencies suggesting that the gemini cationic lipids themselves may be weakly fusogenic.

### 3.3 Multifunctional Cationic Lipids with Additional Functionality

Smart cationic lipids may be designed to possess unique additional functionality such as the ability to promote fusion of the lipid nanocarrier with biological membranes improving transfection ability, Section 3.3.1. They may be able to undergo reversible phase transitions in response to local changes in environment such as pH or temperature, Section 3.3.2. In Section 3.3.3 their ability to assist with targeting of an encapsulated drug to a particular environment in vivo such as the site of bacterial infection, or the tumour microenvironment, is described. Finally, conjugation of a known pharmacophore to a cationic lipid can render such lipids bioactive, Section 3.3.4, with the ability to either enhance the therapeutic effect of a co‐loaded drug or act as a drug in their own right.

#### 3.3.1 Fusogenic Cationic Lipids

Transfer of the gene or drug payload may be facilitated by fusion of the lipid nanocarrier with the external plasma membrane,^[^ ^88^ ^]^ and potentially with internal organelle membranes for example to facilitate lyso‐endosomal escape.^[^ ^89^ ^]^ Fusogenicity is, therefore, an important property of the lipid nanocarrier. Typically, fusogenicity of transfection formulations has been improved via doping with the neutral lipid DOPE, which contains two single, unsaturated oleoyl hydrocarbon chains known to increase the fluidity of the membrane and potentially increase fusion with cellular membranes.^[^ ^90^ ^]^ The small headgroup and presence of unsaturation in the DOPE hydrocarbon chains is associated with a high critical packing parameter (CPP), which promotes high stored curvature elastic stress of the membrane facilitating fusion and endosomal escape. Therefore, in this section, the effect of architecture of the cationic lipid on their fusogenicity is discussed.

**Figure 5:**
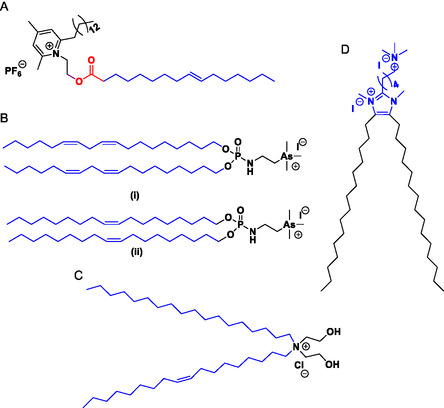
Cationic lipids with improved fusogenic ability A) pyridinium cationic lipid with unsaturated C16 chain B) i) cationic lipophosphoramidate BSV4 (lipid 4) ii) KLN 7 C) Asymmetric S:U cationic lipid D) dicationic imidazolium salt IMeNMe_3_.

The presence of either unsaturation in the hydrocarbon chain (**Figure** **5**A,B),^[^ ^91^, ^92^ ^]^ or asymmetry (Figure 5C)^[^ ^93^ ^]^ in the case of lipids having two chains, Table 2, was shown to increase the fusogenicity. Both strategies will act to increase the effective hydrocarbon chain volume, increasing the CPP which has been shown to promote fusion (and is the rationale behind the use of the high curvature lipid DOPE to facilitate fusion). In the headgroup region, the presence of cationic charge (Figure 5D),^[^ ^94^ ^]^ Table 2, strongly increased the fusogenicity, almost certainly due to increased electrostatic interactions with the typically anionic cell membrane.

#### 3.3.2 Stimuli‐Responsive Lipids

Stimuli responsive cationic lipids are a class of functional excipients which respond to environmental stimuli, such as pH, temperature or light, inducing structural changes in the lipid nanostructure with the subsequent release of the encapsulated cargo. For successful transfection or drug delivery, pH responsive lipids facilitating escape of the lipid and the therapeutic cargo from the low pH environment of the endosomes is critical. The mechanism of endosomal escape is related to the nature of the nanoparticle but can involve burst release due to the proton sponge effect, interactions with the endosomal membrane which can promote endosomal escape or rupturing the endosomal membrane upon swelling due to changes in pH. Thus, engineering cationic lipids with stimuli‐responsive chemical functionalities can improve endosomal escape and further increase the transfection efficiency of the cationic lipid.

**Figure 6:**
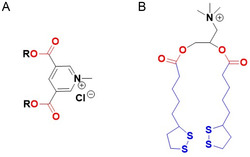
Stimuli‐responsive cationic lipids A) pyridinium cationic lipids, SAINTS B) redox sensitive lipoic acid based cationic lipid (AP1).

A range of studies have investigated the capacity of cationic lipids to exhibit stimuli‐responsive behaviour. Some cationic lipids were rendered pH‐responsive via the introduction of ester‐based linkers (**Figure** **6**A),^[^ ^95^ ^]^ Table 2, which result in cleavage under acidic conditions. This can facilitate endosomal escape for the lipid and the therapeutic cargo via increased interactions with the endosomal membrane. In addition, a redox sensitive cationic lipid was developed by replacing the hydrocarbon chains of a DOTAP molecule with lipoic acid; a constrained dithiolane ring in the lipoic acid undergoes thiol‐disulfide exchange to produce an intermediate which binds DNA in the oxidized state and releases it in the presence of thiols or reductases (Figure 6B).^[^ ^96^ ^]^

#### 3.3.3 Targeting Lipids

**Figure 7:**
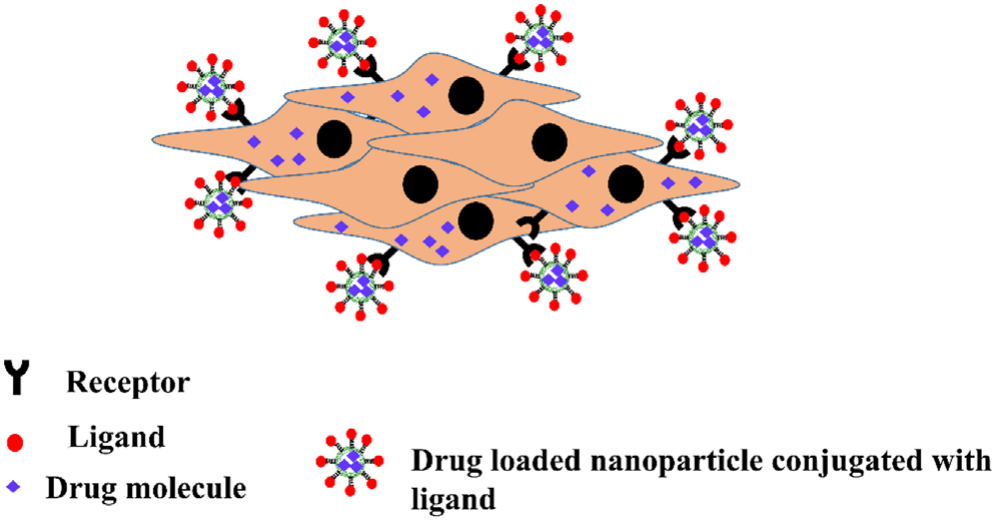
Targeted drug delivery. Selective uptake of nanoparticles tagged with targeting ligand by cells overexpressing the receptors for the conjugated ligand. Cells devoid of the cell surface receptors for the targeting ligand fail to take up the tagged nanoparticles. Adapted with permission.^[^ ^145^ ^]^ Copyright 2017, Elsevier B.V.

Nanoparticles bearing cationic lipids can display increased cellular interactions resulting in non‐specific interactions with cellular membranes in vivo, limiting their efficacy and resulting in undesired side‐effects due to premature release of the drug cargo prior to arriving at the tumour site. To mediate this, active targeting of cationic lipid nanoparticles to cancer cells may be achieved by functionalizing the surface of the nanoparticle using ligands (**Figure** **7**) that bind to specific receptors overexpressed on cancer cell surfaces.^[^ ^97^, ^98^ ^]^ Some major proteins which are widely overexpressed in a variety of tumors and cancer cell lines are fibroblast growth factor receptors (FGFRs),^[^ ^99^ ^]^ epidermal growth factor receptors (EGFRs),^[^ ^100^ ^]^ folate receptors (FR),^[^ ^101^ ^]^ somatostatin receptors (SSTRs),^[^ ^102^ ^]^ as well as members of the superfamily of G‐protein coupled receptors (GPCRs).^[^ ^103^ ^]^ In this regard many research groups have developed molecularly targeting cationic lipids using different small molecule ligands for these receptors overexpressed in cancer. In the following section we summarize active targeting of cationic lipids to cancer cells in various organs including the brain, liver, and lungs by utilizing receptor–ligand interactions. **Note that the presence of cationic charge does not specifically improve the targeting capability. Rather, the cationic charge is used to assist in encapsulation of the negatively charged nucleic acid cargo and the targeting ligand helps to deliver this cargo to a specific cell‐type.**

**Figure 8:**
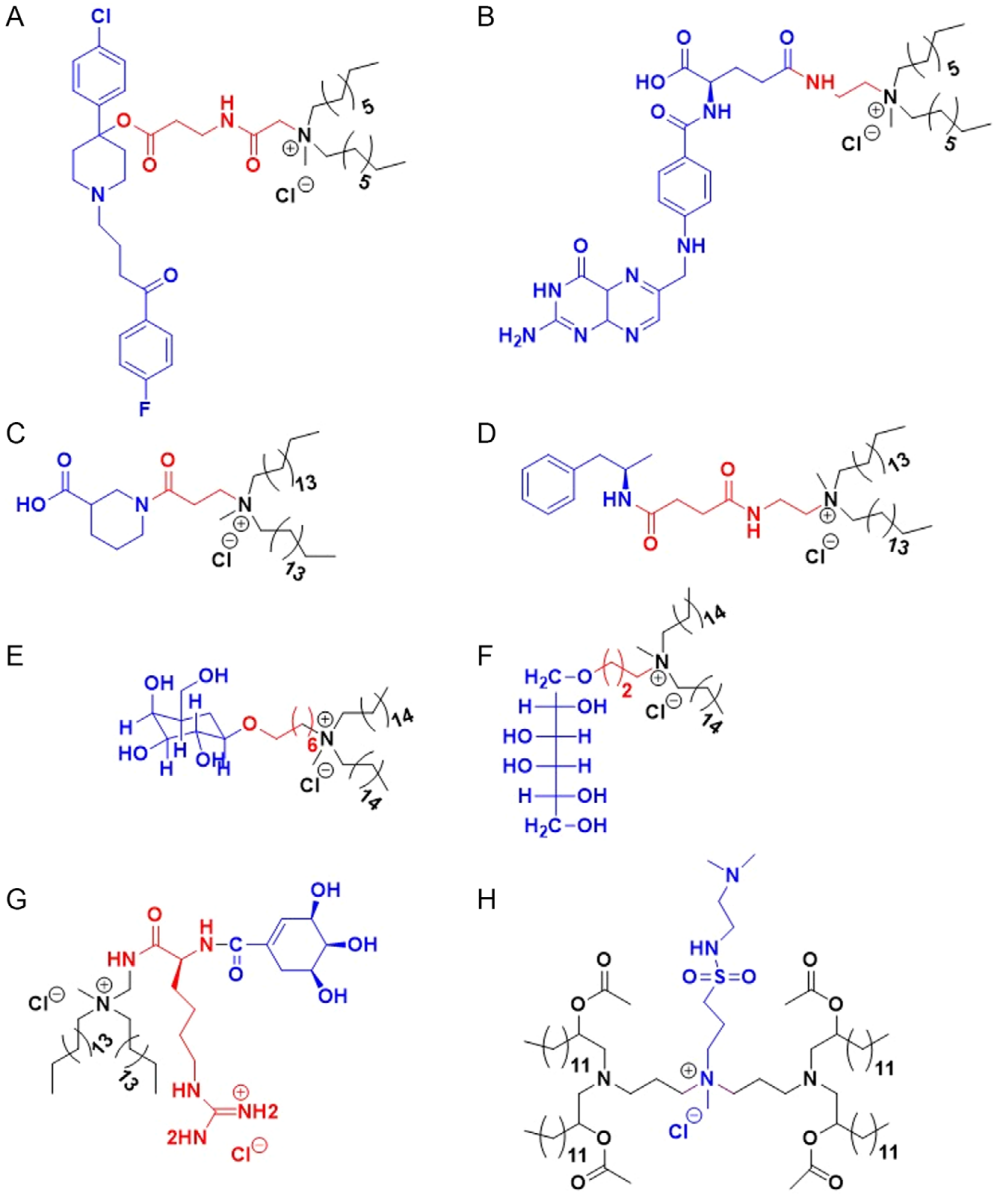
Targeting cationic lipids A) Sigma receptor targeting haloperidol cationic lipid hybrid, HP‐C8. B) Folate receptor (FR) targeting folic acid cationic lipid hybrid, FA 8 C) GABA targeting Nipecotic acid cationic lipid hybrid, NACA D) Blood brain barrier (BBB) targeting *β*‐amphetamine cationic lipid hybrid, 16‐BACL E) Asialoglycoprotein targeting cationic glycolipids with closed sugar head group (lipid 3) F) Asialoglycoprotein targeting cationic glycolipids with open sugar head group (lipid 6) G) Mannose receptor targeting shikimoyl head group (lipid 1) H) Lung targeting, cationic quaternary ammonium sulfonamide amino lipids (CSAL).

General targeting of cancer cells was achieved by utilizing the affinity of various ligands, Table 2, for either sigma receptors^[^ ^104^ ^]^ or FR^[^ ^101^ ^]^ which are overexpressed on tumour cells. An in vitro study demonstrated the specificity of cationic lipids based on the neuroleptic drug haloperidol to sigma receptors (**Figure** **8**A),^[^ ^105^ ^]^ while cationic lipids containing folic acid were successfully used to target the FR (Figure 8B) in in vivo studies.^[^ ^106^ ^]^ In both cases the most effective lipid contained twin saturated, unbranched C8 carbon chains. Receptor–ligand interactions were also successfully used in the development of brain‐targeting cationic lipids. Liposomes consisting of a lipid bearing nipecotic acid (Figure 8C), a competitive inhibitor of the GABA transporter^[^ ^107^ ^]^ expressed on the cell surface of brain tissues, were shown to selectively target a combination of the nucleic acid CDC20 siRNA and the anticancer drug paclitaxel to human neuroblastoma in athymic nude mice.^[^ ^108^ ^]^ Cationic lipids based on *β*‐amphetamine (Figure 8D), a lipophilic psychostimulant with demonstrated ability to penetrate the blood‐brain barrier (BBB) were also shown to accumulate in the brain and deliver the encapsulated cargo paclitaxel and PDL1‐siRNA to the brain improving the overall survivability of orthotopic glioblastoma bearing mice.^[^ ^109^ ^]^ For liver‐targeting the interaction between sugar molecules and the asialoglycoprotein receptors expressed on liver cells was utilized; both closed ring (Figure 8E) and open chain (Figure 8F) sugars were shown to selectively target the pCMV‐SPORT‐*β*gal plasmid DNA to the hepatocytes.^[^ ^110^ ^]^ Immune‐targeting cationic lipids containing either shikimoyl, quinoyl, or mannosyl groups were designed to target the mannose receptor overexpressed on dendritic antigen‐presenting cells. The shikimoyl headgroup (Figure 8G) proved the most effective in selectively delivering the DNA‐vaccine to dendritic cells in vivo and inducing a long‐lasting antimelanoma response.^[^ ^111^ ^]^ Finally, cationic lipids based on pyridinium were successfully used to target the lungs due to the presence of polyamine transporters specific for pyridinium molecules in lung cells.^[^ ^73^ ^]^ CSAL (cationic quaternary ammonium sulfonamide amino lipids) (Figure 8H) also exhibited tendency to accumulate in the lungs.^[^ ^112^ ^]^

Some of these studies investigated changes to the chemical structure of the linker or hydrocarbon chain within the cationic lipid. As with non‐toxic, transfection efficient cationic lipids, and serum compatible lipids, no clear trends were observed here. For cancer‐targeting cationic lipids, the saturated, straight C8 chain was shown in some studies to be most effective for lipids targeting either sigma receptors or FR.^[^ ^105^, ^106^ ^]^ In contrast, twin saturated, straight C16 chains were shown in other studies to be most effective at targeting brain, liver and immune cells.^[^ ^108^, ^109^, ^110^, ^111^ ^]^ While the receptor–ligand interaction has been proved to provide targeting ability to these cationic lipids across a range of tissues, it is clear that no systematic study to date has investigated the effect of the molecular architecture and/or the hydrocarbon chain on efficacy.

#### 3.3.4 Therapeutic Cationic Lipids

##### Anticancer Cationic Lipids

Cellular processes such as cell growth, proliferation, differentiation, motility, and cell death are strictly regulated by complex protein‐controlled signalling networks. The development and progress of cancer can result from the deregulation of multiple signalling pathways due to the accumulation of large genetic alterations in genes coding for these proteins, resulting in aberrant, non‐functional proteins.^[^ ^113^ ^]^ Cancer is, therefore, a particularly important disease target for combination therapy as dysregulation of multiple pathways typically necessitates co‐delivery of multiple drug molecules to the target site of action. In this section we describe the range of strategies used to date to design therapeutic cationic lipids with demonstrated use in combination therapy for the treatment of a range of cancers. Note that, unless otherwise specified, the activity of all the cationic lipids was measured in solution form. Where nanoaggregates of cationic lipids were measured this is specifically mentioned in the text.

Conjugation of a pharmacophore to cationic lipids was shown to improve the anti‐cancer activity for a wide variety of known cancer drugs including the sex hormones, estrogen and progesterone, dexamethasone, hydrocortisone, and stilbenes, Table 2. In addition, lipid conjugation was shown to impart anti‐cancer activity to drugs with no or weak anti‐cancer activity such as the neuropsychotoic drug haloperidol,^[^ ^105^ ^]^ the natural product cordiarimide^[^ ^70^ ^]^ and the small organic molecule benzamide.^[^ ^114^, ^115^ ^]^ For some drugs including progesterone, dexamethasone, hydrocortisone, and haloperidol, the mechanism of action of the anticancer‐cationic lipid hybrid was linked to increased antiangiogenic activity.^[^ ^105^, ^116^, ^117^, ^118^ ^]^ In other cases, conjugation changed the mechanism of action of the drug entirely. For example, lipidation of the weakly anticancer drug cordiarimide imparted inhibition of human DNA ligase 1 which is associated in the development of anticancer drug resistance in tumours.^[^ ^70^ ^]^ Finally, the use of cationic hybrids may assist in cellular uptake of the drug and allows for the co‐encapsulation of anticancer siRNA or shRNA (such as anti‐survivin siRNA) or the small molecule chemotherapeutic (Withaferin).^[^ ^114^, ^115^, ^119^, ^120^ ^]^

**Figure 9:**
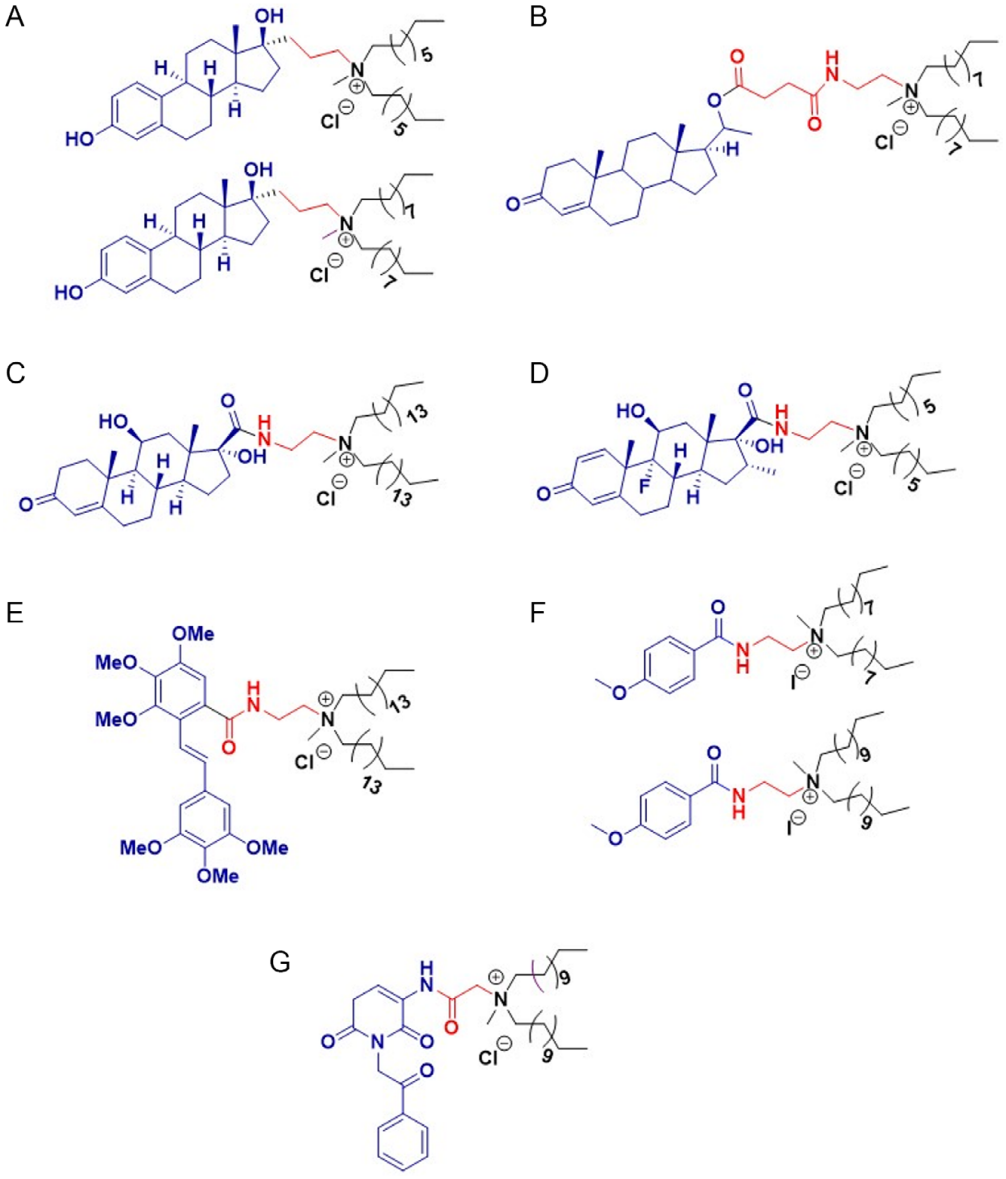
Anticancer cationic lipids A) Estradiol cationic lipid hybrid ESC 8 (top), ESC 10 (bottom) B) Progesterone conjugated cationic lipid hybrid, PR 10 C) Hydrocortisone cationic lipid hybrid, HYC 16 D) Dexamethasone cationic lipid hybrid (DEX 8) E) Methoxy stilbene cationic lipid hybrid HMSC 16 F) Lipobenzamide cationic lipid hybrids (C10M (top), C12M (bottom)) G) Cordiarimide cationic lipid hybrid, NPC 12.

Conjugation to a variety of saturated, unbranched hydrocarbon chains was studied, with typical chain lengths in the range C8–C16. Conjugation of medium length alkyl chains C8–C10 to steroid hormones (estradiol, progesterone, and dexamethasone), haloperidol and emodin was found to result in improved anticancer efficacy.^[^ ^71^, ^105^, ^117^, ^118^, ^121^, ^122^, ^123^ ^]^ For example, the ten‐carbon chain estradiol analogue ESC10 (**Figure** **9**A (bottom)) was found to be 4‐12‐fold more active than the eight‐carbon chain analogue ESC8 (Figure 9A (top)) in breast (MCF‐7, MDA‐MB‐231), melanoma (B16F10), and pancreatic (MIAPaCa‐2) cancer cell lines (Figure 8A).^[^ ^71^, ^121^ ^]^ The C8 analogue ESC8 displayed 47% oral bioavailability in Sprague‐Dawley rats when formulated into solid lipid nanoparticles.^[^ ^124^ ^]^ Among alkyl chain lengths ranging from C6–C18, conjugating progesterone to twin C10 chains (PR 10) (Figure 9B) was shown to result in improved anticancer activity in vitro in a range of cancer cell lines (breast, ovarian, and melanoma of different origin) tested, in vivo studies in melanoma mice model further demonstrated a significant tumor reduction in the group treated with PR 10 compared to the untreated or progesterone treated groups.^[^ ^117^ ^]^ For dexamethasone, DX8 (Figure 9D) the C8 carbon chain analogue, exhibited anticancer activity in lung, breast and melanoma cancer cell lines, which is in alignment with in vivo studies in mice melanoma model. DX10 [Figure A1W) the C10 carbon chain analogue, in combination with the commercial STAT3 inhibitor WP1066 exhibited synergistic anticancer activity in both an in vitro and in vivo melanoma model. In contrast, for hydrocortisone the saturated C16 carbon chain analogue, HYC16 (Figure 9C), was found to have better activity compared to the analogues bearing even numbered carbon chain lengths ranging from C8–C14 in melanoma, breast and lung cancer cell lines, in vivo studies in a melanoma mice model demonstrated tumor regression due to the higher anti‐angiogenic activity of the HYC16 compared to the hydrocortisone.^[^ ^116^ ^]^ Similarly, stilbene (HMSC16) (Figure 9E),^[^ ^120^ ^]^ with C16 alkyl chain length was observed to be most potent anticancer molecule compared to analogues with 8 carbon and 10 carbon chain lengths and is most selective toward the HeLa cancer cell line and MCF‐7 (breast cancer) cell line in vitro.

In case of small organic molecules such as benzamide C10M and C12M (Figure 9F),^[^ ^114^, ^115^ ^]^ cordiarimide NP C12 (Figure 9G),^[^ ^70^ ^]^ saturated, medium alkyl chains in the range C10–C12 were observed to be potent when compared to the short chain and long chain analogues in prostate, liver, and breast cancer cell lines.

##### Antimicrobial Cationic Lipids

The overuse and misuse of antibiotics for decades has led to a global rise in antibiotic resistant microorganisms, with antibiotic resistant bacteria now found in every country in the world. Despite the pressing need for new antibiotic molecules the global pipeline of new antibiotics is very limited. Drugs exhibiting non‐specific action, which act by disrupting the cell membrane, may, therefore, offer a solution. Quaternary ammonium compounds (QACs), as typically found in cationic lipids, are generally used in antiseptics and disinfectants and exert biocidal action by disrupting the phospholipid components of the cytoplasmic membrane.^[^ ^125^, ^126^, ^127^ ^]^ The cationic lipid DODAB, which contains a quaternary ammonium head group, is effective as a bactericide with minimum bactericide concentration (MBC) in the range 3–7.5 μg mL^−1^ (equivalent to 4.7–12.0 μM). It has been demonstrated that positively charged DODAB bilayers in liposomes interact with the negatively charged bacterial cell membrane causing membrane distortion.^[^ ^128^ ^]^ However, due to their non‐specific mechanism of action, quaternary ammonium compounds (QACs) could also be potentially disruptive to mammalian cells. In this regard, some research studies have focussed on the design of cationic lipids which are selectively toxic toward microbes via the selection of suitable head groups.

Anti‐microbial cationic lipids generally contain a quaternary ammonium compound which exerts antimicrobial action by disrupting the external lipid membrane, particularly for Gram‐negative bacteria. The enhanced interaction of cationic moieties with the surface of both Gram‐positive and Gram‐negative bacteria has been well established.^[^ ^129^ ^]^

**Figure 10:**
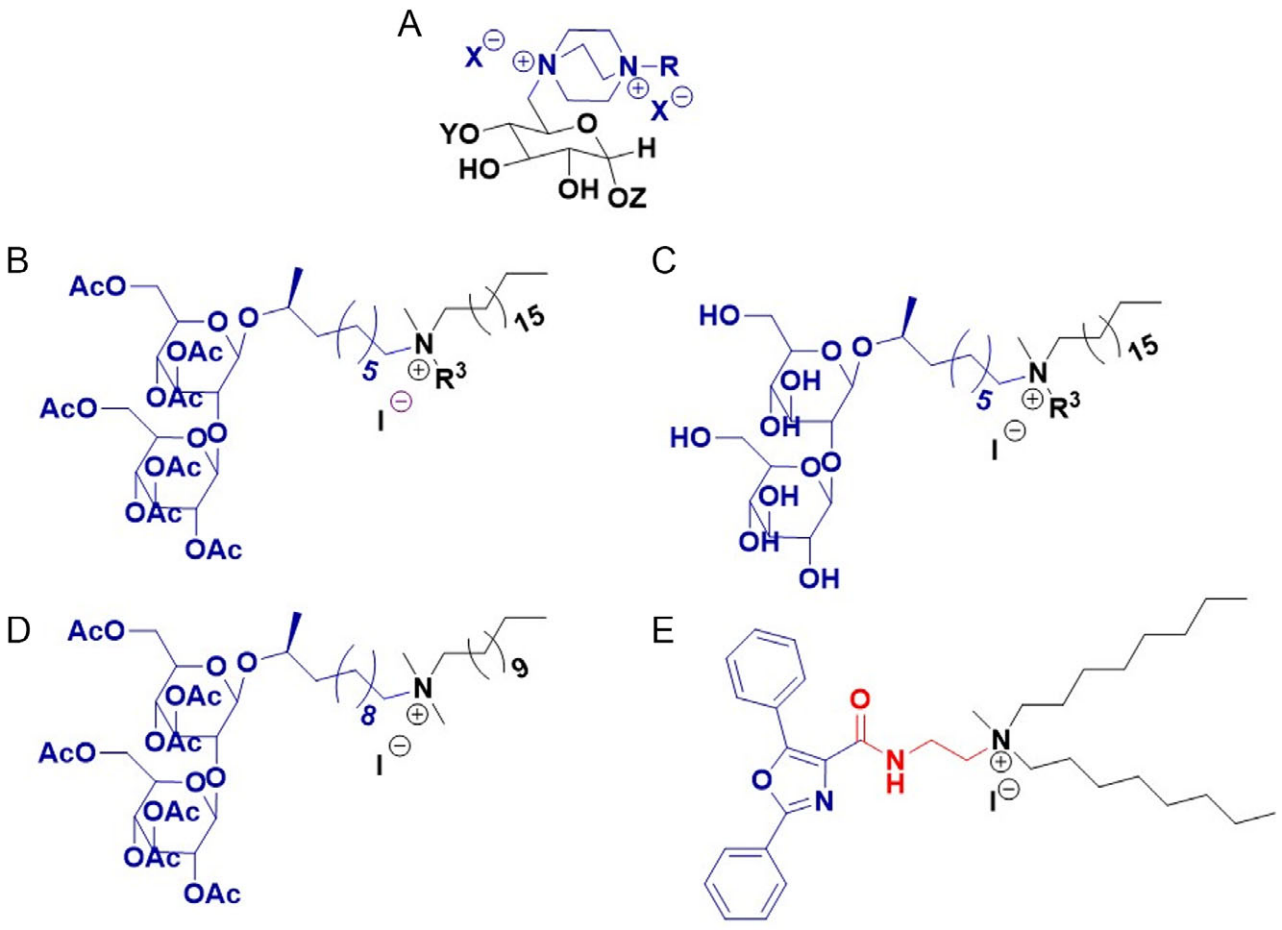
Antimicrobial cationic lipids A) Monosaccharide based cationic lipid hybrids, B) Sophorolipid 11 h (*R* ^3^ = CH_3_), 11i (*R* ^3^ = C_4_H_9_), C) 3 h (*R* ^3^ = CH_3_), 11i (R^3^ = C_4_H_9_), D) Sophorolipid 11d, E) Lipooxazole cationic lipid hybrid, 5c.

Due to their mechanism of action, the charge and/or the inclusion of a quaternary ammonium is the most important molecular feature of the headgroup. A study involving a range of biodegradable carbohydrates, Table 2, including starch, cyclodextrin, and monosaccharide glycosides showed that cationic lipid hybrids based on starch were 5 × 10^4^ times more antimicrobial than the cyclodextrin or monosaccharide analogues due to the ability of the starch moiety to accommodate more positively charged DABCO units (**Figure** **10**A).^[^ ^130^ ^]^

In terms of the alkyl chain, lipids with moderate chain length (C8–C11) were generally associated with lower MICs against bacteria and fungi, Table 2, although a study on the antimicrobial efficacy of sophorolipids (Figure 10B–D) found the C18 analogues to be most effective in inhibiting the growth of some strains including the Gram‐positive bacterial strains *S. aureus* and *B. subtilis*.^[^ ^131^, ^132^ ^]^ We note that the self‐assembly behaviour of the antimicrobial cationic lipid hybrids was rarely characterised, although one study found that it was the ability of sophorolipids to form micelles which enhanced their antimicrobial activity.^[^ ^132^ ^]^

An oxazole cationic lipid hybrid (Figure 10E) with moderate carbon chain length C8^[^ ^133^ ^]^ and benzamide cationic lipid hybrid with odd carbon chain lengths (C9 and C11)^[^ ^134^ ^]^ (Appendix Figure A1Z(ii)) were observed to be active against *C. albicans*. Both the oxazole and benzamide cationic lipid hybrids exhibited selectivity toward microbes while remaining non‐toxic to the mammalian cell lines and erythrocytes at MICs. Thus, unlike first generation cationic surfactants such as DODAB, new generation antimicrobial cationic lipids are selectively toxic toward microbes while unharming mammalian cells at the doses effective for antimicrobial activity.

##### Antioxidant Cationic Lipids

**Figure 11:**
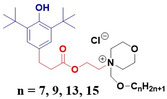
Antioxidant cationic lipid, PPMA‐15 (*n* = 15).

The positive charge of cationic lipids is known to trigger intracellular reactive oxygen species leading to cell necroptosis.^[^ ^67^ ^]^ This effect may be reduced by synthesizing cationic lipid hybrids bearing functional groups such as phenol (**Figure** **11**) with intrinsic antioxidant properties,^[^ ^135^ ^]^ Table 2.

Modifications to the hydrocarbon chain architecture of these lipids was shown to effectively increase their antioxidant efficacy. Specifically, the increased lipophilicity associated with longer hydrocarbon chains was shown to drive increased partitioning of these lipids into the erythrocyte membrane increasing the antioxidant activity.^[^ ^135^ ^]^ While this effect was also associated with increased hemolysis of the cell, the dose required for antioxidant activity was generally lower than that at which hemolysis was observed.^[^ ^135^ ^]^

## 4 Advances in Cationic Lipid Design: A Detailed Overview

In Section 3 we provided a general overview, summarizing some observed trends between the chemical structure of cationic lipids and their toxicity, transfection efficiency, and therapeutic efficacy. The relationship between the chemical structure and additional lipid functionality such as fusogenicity and stimuli‐responsive nature was also described. In the following section additional details are provided on the studies and lipids summarized in Section 3. Due to the wealth of detail in this section, it is designed for the reader seeking additional information on specific cationic lipid classes summarised in Section 3 to be able to obtain this directly from the corresponding sub‐section in Section 4.

### 4.1 Improved Biocompatibility and Transfection Efficiency

#### 4.1.1 Charge Delocalization

The first step toward reducing the toxicity involved replacing the aliphatic head groups with heterocyclic systems; as the quaternary ammonium is part of the heterocyclic ring, the positive charge on the nitrogen atom is spread throughout the ring due to resonance effects. Initially the biodegradable **imidazolium**‐based cationic lipids DMTIM [1‐[2‐(tetradecanoyloxy)ethyl]‐2‐tridecyl‐3‐(2‐hydroxyethyl)imidazolinium chloride], DPTIM [1‐[2‐(Hexadecanoyloxy)ethyl]‐2‐pentadecyl‐3‐(2‐hydroxy‐ethy1) imidazolinium chloride] (Figure A1A) and DOTIM [1‐[2‐(9(*Z*)‐Octadecenoyloxy)ethy1]‐2‐(8(*Z*)‐heptadecenyl)‐3‐(2‐hydroxyethyl)imidazolinium chloride] (Figure 3A(i)) were synthesized with quaternary nitrogen incorporated into the imidazolium ring.^[^ ^72^ ^]^ The hydrocarbon chains are unequal in length with the longer chain attached to N1 position via ethylene spacer and one carbon shorter chain is attached to the C2 position of the imidazolium ring. For in vitro studies imidazolinium cationic lipids were formulated along with the helper lipid DOPE (1:1) while for in vivo studies they were formulated with cholesterol. In vitro transfection efficiency of the imidazolium lipids followed the order DMTIM with myristoyl (singly unsaturated, straight C14 (14:1) acyl chain) > DOTIM with oleoyl (singly unsaturated, straight C18 (18:1) acyl chain) > DPTIM (fivefold lower) with palmitoyl (singly unsaturated, straight C16 (16:1) acyl chain). DMTIM:DOPE exhibited twofold higher efficiency than commercial DOTMA:DOPE. For in vivo studies, DOTIM:Chol demonstrated the highest transfection efficiency, followed by DPTIM and DMTIM.

Later, the use of a **pyridinium** heterocyclic ring was explored to obtain a range of biocompatible cationic lipids with improved transfection efficiency. Two libraries of pyridinium cationic lipids, ester series (52), and amide series (54) each featuring saturated acyl chains of varying lengths (C12–C16) were synthesized. The pyridinium cationic lipid 52b (C14 analogue) from the ester series (52) (Figure 3A(ii)) and 54c (C16 analogue) from the amide series (54) (Figure 3A(ii)) when combined with the helper lipid DOPE demonstrated a substantial increase in transfection efficacy which surpassed that of the commercial formulation DMRIE‐C by 2–6 fold.^[^ ^74^ ^]^ The presence of ester and amide groups in the linker was found to contribute to the biocompatibility of these lipids, although amide analogues were observed to be slightly toxic compared to ester analogues. Another pyridinium‐based cationic lipid, 2Oc, (Figure 3A(iii)), designed by replacing the trimethylammonium of DOTAP with a 2,4,6‐trimethylpyridinium ring,^[^ ^73^ ^]^ exhibited improved transfection efficiency with reduced toxicity compared to DOTAP when co‐formulated with helper lipid cholesterol. Furthermore, 2Oc and DOTAP formulations with cholesterol were twice as effective compared to the formulations in combination with the co‐lipid DOPE. In vitro transfection efficiency was observed to be cell‐line dependent while in vivo, 2Oc exhibited higher transfection efficiency compared to DOTAP in malignant NCI‐H23 lung cancer cells grafted mice.

In another case, the inclusion of a guanidinium group in the cationic lipid (Figure 3A(iv)) also resulted in reasonably non‐toxic cationic lipids with 2‐ to 4‐ fold increase in transfection efficiency compared to Lipofectamine when formulated with cholesterol.^[^ ^75^ ^]^ The reduced toxicity was attributed to charge delocalization over the guanidinium moiety. The presence of the guanidinium group can also help in facilitating translocation across the negatively charged cellular membranes as well as promoting endosomal escape thus improving the transfection efficiency. This study additionally demonstrated the importance of the headgroup positive charge in effective transfection; a control molecule lacking a permanent positive charge (Figure A1B) exhibited poor transfection efficiency.^[^ ^75^ ^]^

#### 4.1.2 Conjugation of a Naturally Available Molecule

In contrast, conjugation of a naturally existing molecule to the quaternary ammonium group was also shown to improve the biocompatibility of the cationic lipids. Toward this end a carotenoid molecule was conjugated to a quaternary ammonium cationic lipid (Figure A1C);^[^ ^78^ ^]^ however, although the carotenoid‐based lipoplexes were more biocompatible than DC‐Chol [1,(3*β*‐[*N*‐(*N′*,*N′*‐dimethylamino‐ethane)carbamoyl]‐cholesterol] (up to (+/−) molar charge ratio of 2.5), no improvement in transfection efficiency was observed. Similarly, the conjugation of cholesterol to a tri‐2‐hydroxyethylamine (THEA) head group via an ether linker resulted in the reasonably biocompatible cationic lipid Chol‐THEA (Figure 3B(i)).^[^ ^79^ ^]^ Chol‐THEA:DOPE liposomes exhibited transfection efficiencies better than the commercially available lipids DOTAP, Lipofectamine, and DMRIE‐C in Huh‐7 cells, and similar or higher levels of transfection to these commercially available lipids in MCF‐7 cells. In another study, the naturally occurring molecule lactic acid (1‐hydroxy propyl group) was grafted to a quaternary ammonium bearing twin saturated, straight hydrocarbon chains of varying lengths (C8–C18)^[^ ^80^ ^]^ to obtain biocompatible lipids. The transfection efficiency of these lipids was found to be dependent on the hydrophobic tail; lipid 4 or DMHMAC [*N,N*‐di‐myristyl‐*N*‐(1‐hydroxyprop‐2‐yl)‐*N*‐methylammoniumchloride] (Figure 3B(ii)) bearing twin C14 carbon chains when co‐formulated with cholesterol was found to have the highest transfection efficiency and had similar or better transfection efficiencies than the lipid DHDEAB [*N,N*‐di‐*n*‐hexadecyl‐*N,N*‐dihydroxyethylammoniumbromide] (which is known to have superior transfection efficiency compared to Lipofectamine). Similarly, the nucleobase uracil was grafted into the DOTAP structure to synthesize the biocompatible cationic lipid DOTAU [*N*‐[5′‐(2′,3′‐dioleoyl)uridine]‐*N′,N′,N′*‐trimethylammonium tosylate] (Figure 3B(iii)).^[^ ^81^ ^]^ DOTAU was observed to self‐assemble at room temperature into liposome‐like structures in aqueous solutions. Small angle X‐ray scattering (SAXS) studies confirmed that the п–п stacking interactions result in the close association of the head group with the encapsulated nucleic acid while IR studies confirmed the hydrogen bonding interactions of the uracil (nucleobase) with the adenine of the encapsulated nucleic acid. However, the observed transfection efficiency was lower than Lipofectamine, except at relatively high concentrations of the nucleolipid.

#### 4.1.3 Gemini Cationic Lipids

Gemini cationic lipids are dimeric amphiphiles wherein two cationic amphiphiles are connected into a single molecule at their head groups via a spacer. A variety of gemini cationic lipids have been synthesised based on pyridine^[^ ^74^ ^]^ and cardiolipin.^[^ ^83^, ^136^ ^]^ These gemini cationic lipids were shown to self‐assemble into aggregates at lower concentrations and display increased transfection efficiency compared to their monomeric counterparts, due to their higher charge: mass ratio.^[^ ^74^, ^83^, ^136^ ^]^ Structure–activity relationship studies indicated that the length of the spacer and hydrocarbon chains were shown to dictate the aggregation properties and transfection efficiency. In the case of pyridinium cationic gemini surfactants with non‐polar carbon linkers of varying length (Figure 3C(i)), and hydrophilic linkers based on secondary and tertiary amino group(s) (Figure 3C(ii), A1D(ii)) it was observed that lipids with hydrophilic linkers displayed much lower transfection efficiencies than their related gemini congeners with more hydrophobic linkers.^[^ ^74^ ^]^ The gemini surfactant 29 (two methylene unit spacer) was found to have the highest transfection efficiency for the MDA‐MB‐231 cell line, being two times more efficient than the commercial cationic lipids DMRIE‐C, DOTAP and Lipofectamine. In contrast, a library of similar gemini surfactants containing a disulfide linker (Figure A1D(i and iii)) was observed to be devoid of any transfection ability.^[^ ^74^ ^]^ Cationic cardiolipin analogue (CCLA) (Figure 3C(iii)), a gemini cationic lipid, based liposomes were observed to be less toxic compared to the commercial DOTAP‐based In Vivo GeneSHUTTLE. CCLA liposomes effectively transfected the siRNA both in vitro and in vivo when formulated with DOPE in 1:2 ratio, with an efficacy seven‐fold higher than DOTAP‐based liposomes.^[^ ^83^ ^]^

#### 4.1.4 Modifications to the Hydrocarbon Chain

**Figure 12:**
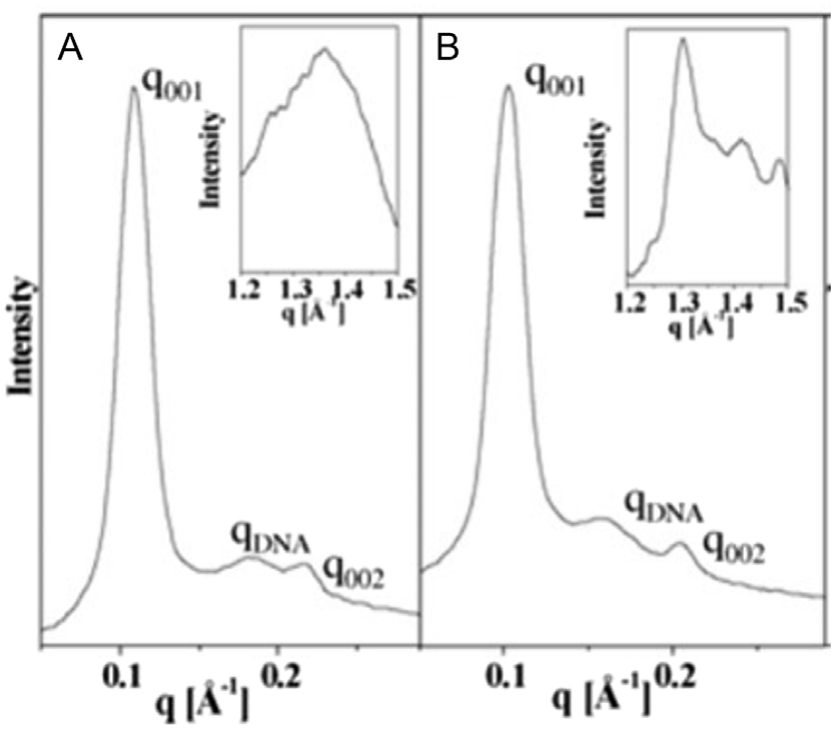
SAXS (small‐angle X‐ray scattering) and WAXS (wide‐angle X‐ray scattering) patterns (inset) of A) DOTAP B) analogue 4. SAXS shows both DOTAP and analogue 4 lipoplexes assume lamellar $L_{\alpha }^{C}$ phase with a variation in DNA packing. WAXS of liposomes shows that DOTAP chains exhibit fluid lamellar phase (L*α*, broad isotropic peak at *q* = 1.35 Å – 1) and analogue 4 chains exhibit gel like (*L*_*β*_, sharp ordered peak at *q* = 1.30 Å − 1) phase at 30 °C. Reproduced with permission.^[^ ^82^ ^]^ Copyright 2006, American Chemical Society.

It was demonstrated that increasing the stability of the self‐assembled nanoparticle by chemical modifications to the hydrocarbon chain resulted in improved transfection efficiency, potentially due to reduced leakage of the encapsulated cargo. Replacement of the cis‐double bonds of the oleate chains in DOTAP with single C≡C (carbon–carbon triple bond) yielded dialkynoyl DOTAP congeners ( Figure A1E).^[^ ^82^ ^]^ The position of the triple bond influenced the rigidity of the lipoplex membrane with the lipid analogue 4 (Figure 3D(i)) bearing the triple bond toward the end of the chain (between carbon 14 and carbon 15) being more stable compared to analogue 2 with the triple bond at the start of the chain, (between carbon 4 and carbon 5). Lipid 4:cholesterol lipoplexes exhibited 2–3 times higher transfection compared to DOTAP: cholesterol lipoplexes. SAXS and wide angle X‐ray scattering (WAXS) studies (**Figure** **12**) indicated that the analogues readily formed lamellar structures and the type of unsaturation (i.e., triple bonds) and position of the triple bond in the chain influenced the rigidity of the lipoplex membrane. At 30 °C, a fluid lamellar (*L*_α_) phase was observed with DOTAP, while a gel‐like (*L*_β_′) phase was observed with analogue 4.

#### 4.1.5 Shape of the Cationic Lipid

Besides the nature of the constituent chemical groups, the molecular architecture or shape of the cationic lipids can also dictate the transfection efficiency of the molecule. For example, among the three pyridinium cationic lipids, lipid 8 (Figure A1F(i)), 9 (Figure 3D(ii)), and 10 (Figure A1F(ii)), lipid 9 with a truncated cone shape exhibited the highest transfection efficiency, potentially due to the higher surface curvature and smaller size of the liposomes.^[^ ^74^ ^]^

### 4.2 Cationic lipids with Enhanced Serum Compatibility

Serum compatibility, to determine whether the cationic lipids can successfully transfect cells in the presence of serum, is typically assessed by measuring expression levels of the reporter gene transfected by cationic lipids in the presence of serum.

A range of gemini cationic lipids based around an aromatic backbone exhibited improved transfection efficiencies including in the presence of serum (Figure A1G(i),(iii), monomer Figure A1G(ii)). Two classes, one bearing an oxyethylene spacer (Figure A1G(i)),^[^ ^86^ ^]^ with spacer length ranging from 1–3 units, and unbranched, saturated C14 and C16 alkyl chains; the other a polymethylene spacer (Figure A1G(iii)), with spacer length in the range 3–12 units and straight, saturated alkyl chains C12, C14, and C16 were developed.^[^ ^85^ ^]^ For gemini lipids bearing a polymethylene spacer, the lipid 5c (Figure 4A(i)) with tetradecyl (C14) chains and a pentamethylene spacer (5 units) showed the highest gene transfection efficacy. For those lipids with an oxyethylene spacer, the effect of the spacer length on transfection was dependent on the hydrocarbon chain length. Lipid analogues with C14 chains displayed an increase in transfection efficiency with increasing spacer length; the opposite trend was observed in lipids with C16 chains. Gemini lipids with an oxyethylene spacer bearing C16 chains showed better gene transfection activities compared to their analogues bearing C14 chains. The effect of the chemical identity of the spacer, and the length of both the spacer and the hydrocarbon chain on transfection efficiency was therefore complex. Neat gemini cationic lipids were found to have poor transfection capabilities which improved when the lipids were formulated with DOPE. A formulation consisting of lipid 4c (Figure 4A(ii)) with a three oxyethylene unit spacer and DOPE was found to be the most active with reasonable transfection efficiencies observed up to 50% serum.

Cholesterol‐based gemini cationic lipids (Figure A1H(i)), synthesized by conjugating two monomeric units (Figure A1H(ii)) of cholesterol based amphiphiles connected to a quaternary ammonium head group via a polymethylene spacer with spacer length ranging from 3 to 12 units,^[^ ^87^ ^]^ also exhibited serum compatibility. The transfection efficiency changed with spacer length, both in the presence and absence of serum. The optimum spacer length was found to be a five carbon pentanediyl group [–(CH_2_)_5_–]. The transfection efficiency of the gemini lipid 2c (Figure 4B), having a five carbon pentanediyl spacer, was nearly two times more effective than formulation E, one of best‐known commercially available transfecting reagents. At present the precise mechanism by which varying the length of the spacer impacts protein binding, serum compatibility and transfection efficiency has not been determined.

**Figure 13:**
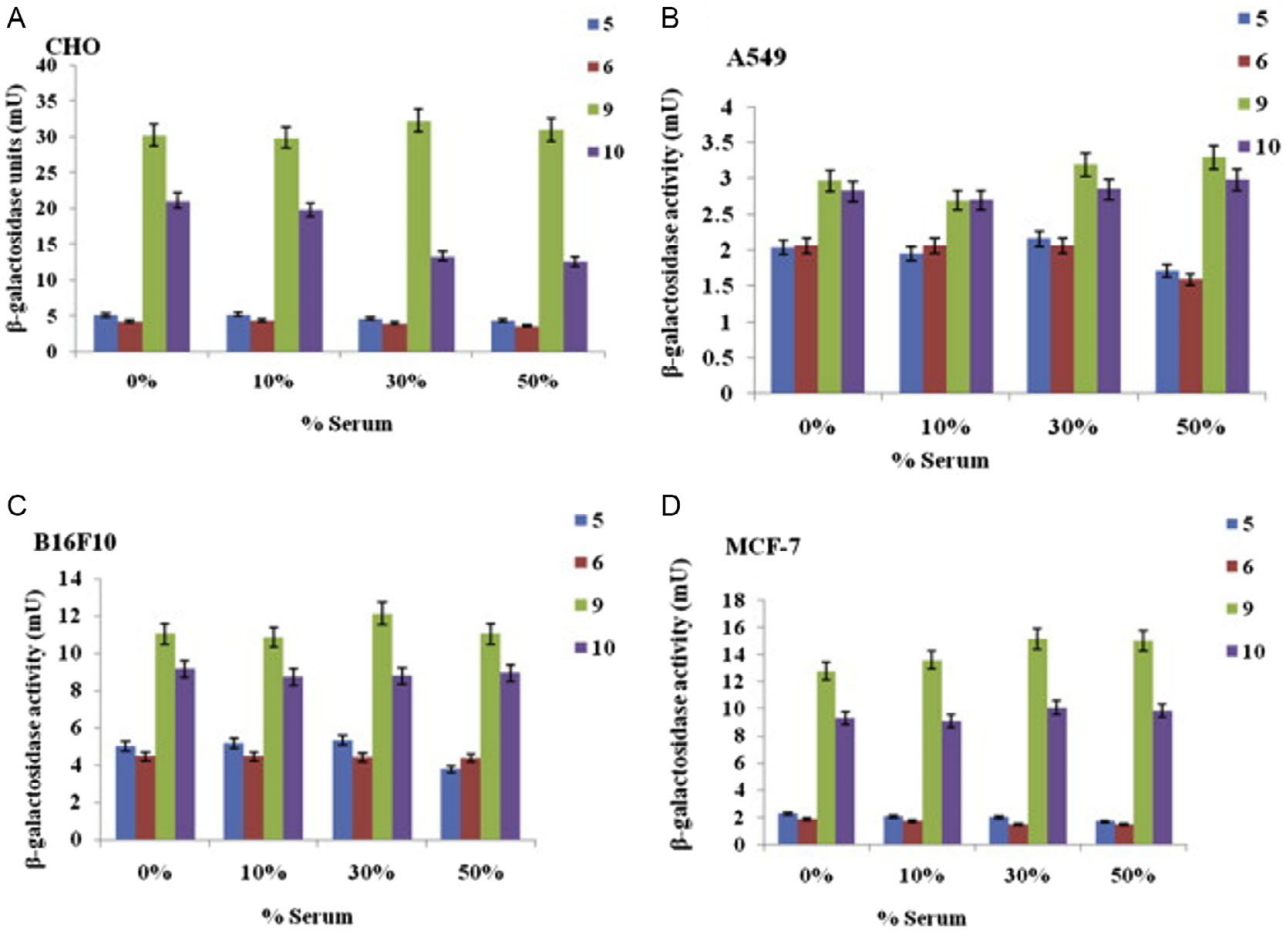
In vitro transfection efficacies of the cationic lipids 5, 6, 9 and 10 in the presence of increasing concentrations of added serum of lipid:DNA (8:1) complexes prepared using *p*CMV‐*β*‐gal‐SPORT reporter gene were evaluated in: A) CHO, B) A‐549, C) B16F10, and D) MCF‐7 types of cells. Reproduced with permission.^[^ ^77^ ^]^ Copyright 2013, Elsevier Masson SAS.

Cationic lipids based on benzothiazole,^[^ ^77^ ^]^ tocopherol,^[^ ^76^ ^]^ and Tris base^[^ ^84^ ^]^ were all reported to have better serum compatibility owing to the presence of polar groups such as hydroxy and amine functional groups in the chemical structure. Benzothiazole analogues bearing hydroxy (Lipid 5, Lipid 6) and amine groups (Lipid 9, Lipid 10) on the benzothiazole ring (Figure A1I) effectively transfected reporter genes, including in the presence of serum up to 10% (v/v) (**Figure** **13**). Lipid 9 (saturated C16 analogue) and lipid 10 (saturated C18 analogue) bearing amine groups (Figure 4C(i) and (ii)) were found to display transfection efficiencies comparable to or greater than that of the commercial gene transfection agent Lipofectamine and displayed the highest serum compatibility.

Tocopherol cationic lipids containing oxyethylene spacers and hydroxyethyl moieties in the head group also exhibited good serum compatibility (Figure A1J(ii)).^[^ ^76^ ^]^ Lipid 5 (Figure 4D), which contains three —OH groups, had the highest serum compatibility. The transfection efficiency also increased with the number of hydroxyl groups and was highest for lipid 4 (2 —OH groups) and lipid 5 (3 —OH groups) with transfection efficiencies either similar to, or 2–4 times higher than, Lipofectamine, depending on the cell line. The extremely low transfection ability displayed by the control lipid 6 (Figure A1J(i)), where all three hydroxyl groups of lipid 5 are replaced with methoxy groups, signifies the importance of hydrogen‐bonding interactions in the stability of the lipoplexes where the hydroxyl groups on the polar head group interact with the phosphate backbone in the nucleic acid promoting the formation of stable lipoplexes.^[^ ^76^ ^]^ Lipids containing hydroxyl groups may facilitate cellular uptake of the lipoplexes thus contributing to their higher transfection efficiencies.^[^ ^76^ ^]^ This may be due to the hydrophilic screen formed by the hydroxyl groups on the surface of the nanoparticles which sterically hinders the interaction with other nanoparticles or serum proteins (as observed with PEG coated nanoparticles)^[^ ^137^ ^]^ thus reducing formation of a protein corona and allowing the positively‐charged nanoparticle to interact with the negatively charged glycocalyx. Covalent grafting of a Tris‐base in the headgroup region to twin saturated C16 aliphatic chains resulted in the cationic lipid, Tris‐lipid 1 (Figure 4E), which not only had high serum compatibility, but actually demonstrated improved transfection efficiencies 2‐ to 3‐fold higher in up to 50% (v/v) serum concentrations, compared to serum‐free conditions.^[^ ^84^ ^]^ Dynamic light scattering (DLS) studies indicated that the Tris‐lipid 1 lipoplex size did not increase upon incubation for over 4h in 10% FBS (Fetal bovine serum) suggesting protein interactions have been reduced. Control experiments with lipid 2 (where the Tris group was replaced with an ethanolamine group containing a single hydroxyl) and lipid 3 (where the hydroxyl groups were replaced with methoxy groups) confirmed the importance of hydroxy groups in serum compatibility. Thus, the high serum compatibility of the Tris‐lipid 1 was attributed to the enhanced surface charge shielding by the three hydroxyl headgroups.^[^ ^84^ ^]^

In another study a series of *α*‐tocopherol based cationic lipids were synthesised; these lipids bear identical head group, hydrophobic chain, and linker except for the backbone region linking the hydrophobic portion to the hydrophilic head domain (Figure 4F).^[^ ^138^ ^]^ It was observed that lipid 1 (with a non‐glycerol backbone) showed higher stability and gene transfer efficacies compared to lipid 2 (with a glycerol backbone) across various cell lines (CHO, B16F10, HepG2, MCF‐7, and A549). Fluorescence microscopy studies of lipoplexes of these lipids (either rhodamine‐labelled or containing GFP (green fluorescent protein) encoding plasmid DNA) demonstrated higher cellular uptake and significantly higher expression of GFP for lipoplexes of lipid 1 compared to lipid 2. Lipid 1 was found to be highly serum compatible even at the highest serum concentration studied (50%).

The addition of hydroxyl groups to the polar headgroup was again used as an effective strategy, in combination with the introduction of a hybrid ether ‐*β* hydroxy‐triazole linker, with the resulting *α*‐Tocopherol cationic lipid hybrids (Lp1–Lp3) shown to be serum compatible due to efficient screening of the surface charge.^[^ ^139^ ^]^ The lipids Lp1 (Figure 4G(i)) and Lp2 (Figure 4G(ii)), both bearing a Tris (2‐hydroxyethyl) ammonium head group, were found to be highly serum compatible at all serum concentrations studied. In contrast, Lp3 (with hydroxyl groups replaced by ethyl groups in the headgroup region) (Figure A1K) showed only moderate transfection in the presence of added serum.

### 4.3 Multifunctional Cationic Lipids with Additional Functionality

#### 4.3.1 Fusogenic Cationic Lipids

A relatively small number of studies have focussed on the impact of the cationic lipid architecture on the fusogenic nature of these lipids. These studies suggest that the chemical structure of both the headgroup and the tail portion impact the fusogenicity of cationic lipids. Several studies investigated the role of unsaturation in the hydrocarbon chains on transfection efficiency. A library of pyridinium cationic lipids, with an amide and ester‐based linker, were synthesized where a saturated, unbranched C14 chain was attached to the C‐2 position of the pyridine ring and a second hydrocarbon chain, varying in length and saturation, was attached via either an amide or ester linker to the ring nitrogen “N” atom (Figure A1L).^[^ ^91^ ^]^ The chemical identity of the linker and hydrocarbon chain were both demonstrated to impact the transfection efficiency. In terms of the linker, analogues with an amide linker performed better than those with an ester linker. In terms of the hydrocarbon chain, analogues with either a saturated C16 or singly unsaturated (16:1) C16 unbranched acyl chain (Figure 5A) exhibited the highest transfection efficiency. For unsaturated analogues, the trans isomer pyridinium lipids performed better compared to the cis isomer and exhibited better transfection efficiency in the presence of high serum concentrations compared to Lipofectamine.^[^ ^91^ ^]^

The introduction of unsaturated chains was also used as an effective strategy to impart fusogenicity with the cationic lipid BSV4 (lipid 4) (Figure 5B(i)) containing two doubly unsaturated (18:2) linoleic lipid chains conjugated to a trimethyl arsonium head group via a phosphoramidate ethylene spacer.^[^ ^92^ ^]^ Anisotropy studies and FRET studies indicated an increase in the fluidity and fusogenicity of self‐assembled BSV4 liposomes compared to the monounsaturated (18:1) analogue KLN47 (Figure 5B(ii)). Transfection studies on BSV4 lipoplexes demonstrated significant expression levels of luciferase (reporter gene) with expression levels almost equal to Lipofectamine lipoplexes depending on the cell type and charge ratio used.

**Figure 14:**
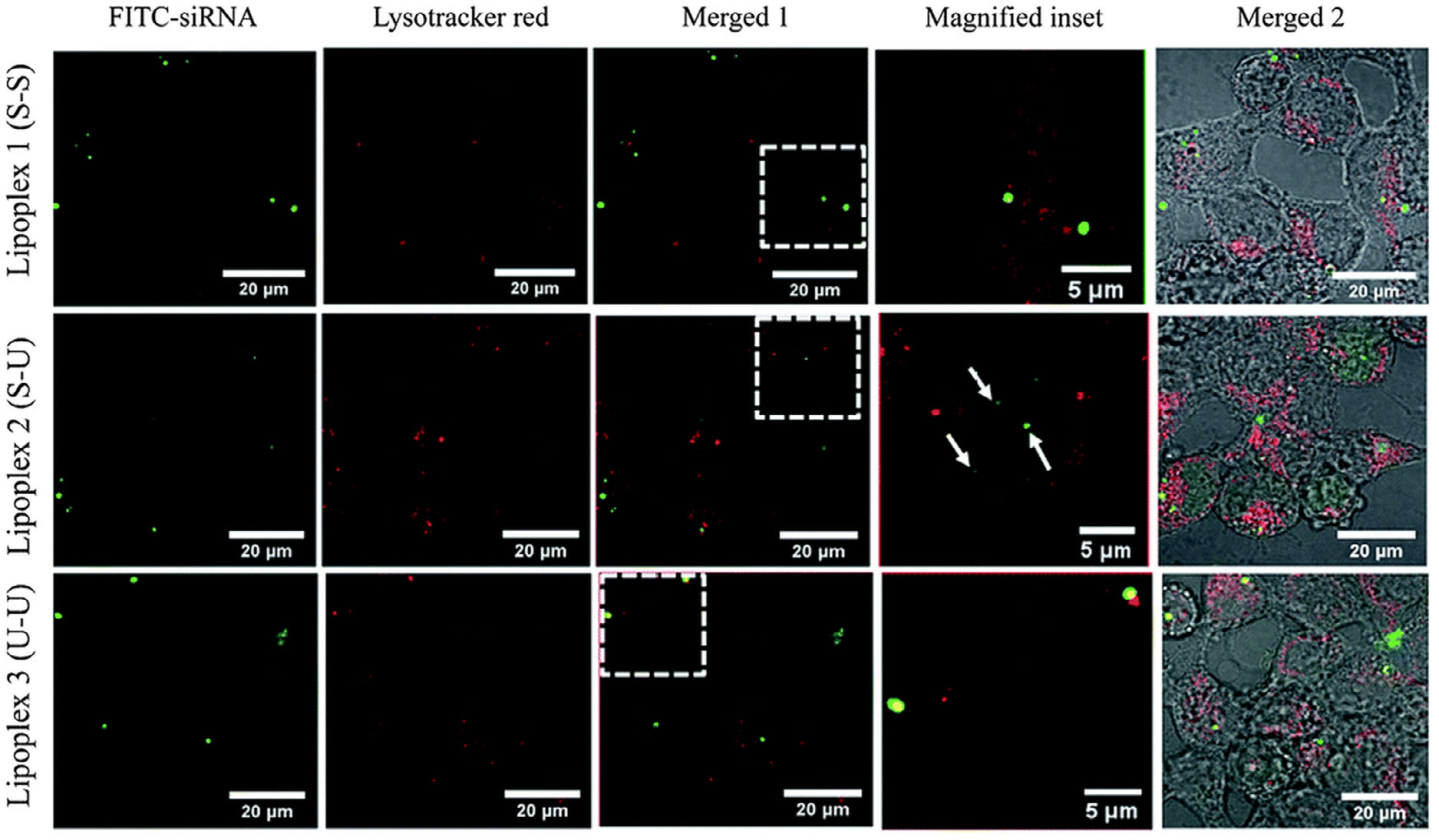
Endosomal escape of U–U, S–U, and S–S lipoplexes studied in HEK293 cells using confocal laser scanning microscopy. Green dots in FITC‐siRNA panel indicate lipoplexes ladened with FITC tagged siRNA and red color in lysotracker red panel indicates lysosomes trailed with lysotracker. Merged 1 panel shows both the lysosomes and FITC siRNA, arrows in the magnified inset panel point to the localization of the lipoplexes in the nucleus. Reproduced with permission.^[^ ^93^ ^]^ Copyright 2016, The Royal Society of Chemistry.

In another study the role of both unsaturation and asymmetry in the hydrophobic chain on the fusogenicity of liposomes was systematically investigated.^[^ ^93^ ^]^ The fusogenic properties of three different types of cationic lipids were investigated using fluorescence‐activated cell sorting (FACS) and epifluorescence microscopy (**Figure** **14**): S–S comprising two saturated, unbranched stearyl chains (18:0) (Appendix Figure A1M(i)), S–U with an asymmetric hydrophobic core comprising both a saturated stearyl (18:0) (Figure 5C) and an unsaturated oleoyl (18:1) unbranched chain, and U–U having two unsaturated, straight oleoyl chains (18:1) (Appendix Figure A1M(ii)). Transfection studies showed that liposomes formed from the asymmetric lipid S–U (with cholesterol in 1:1 ratio) displayed a superior transfection profile (89% cellular uptake) compared to its symmetric counterparts, with 66% uptake observed for the saturated lipid S–S and 70% uptake for the unsaturated lipid U–U.

**Figure 15:**
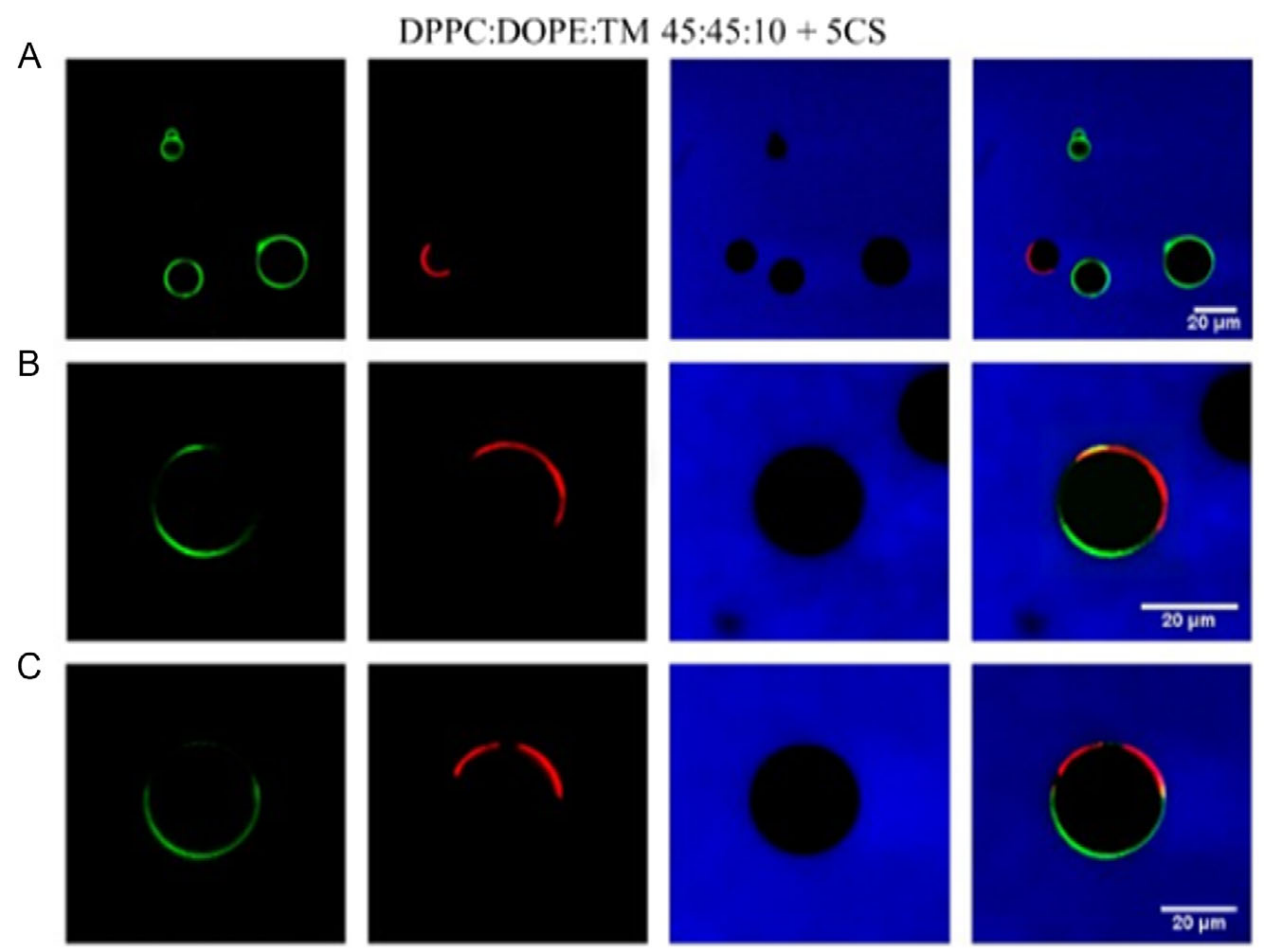
IMeNMe3 vesicle fusion experiments—confocal fluorescence microscopy cross‐sectional images. First column: GUVs labelled by NBDDHPE, second column: GUVs labelled by N‐Rhodamine‐DHPE; Third column: Buffer marked with Atto‐647; Fourth column: Combined image. A) GUVs without IMeNMe3, green immobilized on solid support; target GYVs labelled red—added in bulk to the immobilized green GUVs. B) GUVs with IMeNMe3 labelled green and immobilized on solid support; target GUVs labelled red—added in bulk to the immobilized GUVs. C) GUVs with IMeNMe3 labelled green—added in bulk; target GUVs labelled red—added in bulk. The scale bar of the images corresponds to 20 μM. Reproduced with permission.^[^ ^94^ ^]^ Copyright 2020, Wiley‐VCH GmbH.

Finally, the importance of the quaternary ammonium group on fusion was shown in a study involving the dicationic imidazolium salt, IMeNMe_3_ [1,3dimethyl‐4,5‐dipentadecyl‐2‐(5‐(trimethylammonio)pentyl)imidazolium iodide] (Figure 5D) which was synthesized by introducing the quaternary ammonium salt at the C2 position of the imidazolium core.^[^ ^94^ ^]^ The fusogenic properties of the lipid were investigated using fluorescence microscopy (**Figure** **15**). Transfection studies showed that DPPC: DOPE (1:1) liposomes supplemented with 20% IMeNMe_3_ were successful in transfecting HeLa cell lines. In contrast, the lipid IMeH (1,3‐dimethyl‐4,5‐dipentadecylimidazolium) (Figure A1N) which lacks a quaternary ammonium group at the C‐2 position, failed to transfect HeLa cells when doped into liposomes indicating the importance of the quaternary ammonium group at the C‐2 position. However, transfection efficiencies of IMeNMe_3_ were lower compared to Lipofectamine mediated transfection, potentially due to the lack of unsaturation in the hydrocarbon chains.

#### 4.3.2 Stimuli‐Responsive Lipids

A library of pyridinium cationic lipids SAINTS (Synthetic Amphiphiles Interdisciplinary) (Figure 6A) were synthesized by introducing two hydrolytically cleavable ester‐based linkers between the hydrophilic head group and hydrophobic tail group.^[^ ^95^ ^]^ It was hypothesized that the ester linkers would be cleaved under acidic conditions, facilitating endosomal escape. However, hydrolysis experiments revealed that the rate of ester hydrolysis was significant at higher pH (compared to the acidic pH in endosomes) and NMR experiments confirmed that the hydrolysis of the first ester group hinders the hydrolysis of the second ester group due to the formation of zwitterionic structures. Despite this drawback, the cationic lipids with unsaturated chains were observed to have better transfection efficiencies compared to the saturated analogues in this library. Unsaturated lipids were also observed to form bilayer vesicles upon hydration and sonication.

A redox sensitive cationic lipid, AP1 (Figure 6B) was developed by replacing the aliphatic tails of DOTAP with lipoic acid.^[^ ^96^ ^]^ Lipoic acid harbours a constrained dithiolane ring which is thermodynamically unstable and undergoes thiol‐disulfide exchange at alkaline pH (catalyzed by DTT (Dithiothreitol)) forming the polymer AP2 (Figure A1O). In the oxidized state AP2 binds DNA; intracellular reduction of AP2 in the presence of thiols or reductases then releases the DNA in a stimuli‐responsive manner. In vitro transfection studies revealed that AP2‐DNA complexes achieved transgene expression levels several fold higher than the commercial cationic lipid DOTAP indicating the role of stimuli‐responsive cargo release on transfection efficiency. Flow cytometry studies revealed that although cellular uptake of the AP2‐pDNA complex was lower than that of the DOTAP‐pDNA complex, the selective redox‐linked release mechanism exhibited by AP2 facilitated increased expression of the reporter genes generated by the p‐DNA compared to DOTAP mediated p‐DNA transfection. Thus, the presence of reducible groups such as —S—S— bonds (lipid AP1) and polar groups such as hydroxy groups have been shown to facilitate endosomal escape due to their susceptibility to lower pH values in the endosomes (Figure A1O).

**Figure 16:**
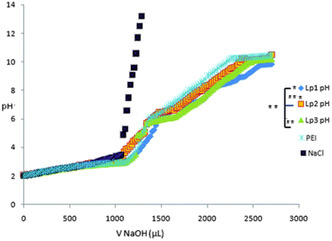
Acid‐base titration profiles of 25 kDa cationic polymer PEI, 150 mM NaCl and lipid solutions. Lipids or PEI (0.050 mmol of cationic lipids Lp1, Lp2, and Lp3) were first treated with 1 N HCl to adjust the pH to 2.0, and then the solution pH was measured after each addition of 20 mL of 0.1 N NaOH solution. *P < 0.05, **P < 0.01, ***P < 0.001. Reproduced with permission.^[^ ^139^ ^]^ Copyright 2018, The Royal Society of Chemistry.

The serum compatible tocopherol cationic lipid analogue Lp2 (Section 3.2, Figure 4G (ii)) was observed to additionally possess stimuli responsive behaviour owing to the presence of a triazole ring in the linker and the Tris (2‐hydroxy ethyl) quaternary ammonium head group.^[^ ^139^ ^]^ Acid‐base titration studies (**Figure** **16**) revealed that analogue Lp2 with pKa 5.5 has good buffering capacity, almost equal to the commercial polyethylenimine (PEI) cationic polymer. Hydrolysis studies indicated significant degradation of lipid Lp2 at acidic pH (70% at pH 5.5 and 100% degradation at pH 4.5) which was attributed to the hydroxyl groups in the head group region and the triazole moiety in the linker which could potentially facilitate early endosomal escape for the lipid Lp2.

#### 4.3.3 Targeting Lipids

Cationic lipid nanoparticles are often employed to deliver a nucleic acid cargo to diseased cells. Non‐functionalized cationic nanoparticles can exhibit non‐specific cellular uptake resulting in unwanted side effects due to non‐targeted drug delivery. Therefore, to facilitate active targeting of these nanoparticles, cationic lipids may be covalently conjugated with targeting ligands. For this class of cationic lipids, the positive charge aids in encapsulation of the negatively charged nucleic acid cargo, while the targeting ligand facilitates the targeted delivery of the cargo. A range of cationic lipids reviewed herein have been successfully targeted to tissues including cancer, brain, lung, and liver cells by utilizing a wide range of receptor–ligand interactions.

##### Cancer‐Targeting Cationic Lipids

Sigma (σ) receptors,^[^ ^104^ ^]^ which are overexpressed in rapidly proliferating cells and found in a range of cancers originating in the brain, skin, breast, prostate, and lungs, can act as a general target for cancer treatments. The neuroleptic drug, haloperidol, has high affinity for sigma receptors. A library of haloperidol based cationic lipids^[^ ^105^ ^]^ containing saturated, unbranched carbon chains of lengths, C4, C8, C12, and C16, (Figure A1P) were synthesized by incorporating the drug molecule within the headgroup region conjugated to a quaternary ammonium cationic lipid via a tertiary hydroxy group. HP‐C8 (Figure 8A), an analogue with twin C8 carbon chains, demonstrated sigma receptor‐assisted antiproliferative activity against the breast cancer cell lines MCF‐7, MDA‐MB‐231, ZR‐75‐1 and the melanoma cancer cell line B16F10; the non‐cancer cell lines COS‐1 and HEK‐293 remained unaffected. The specificity of this lipid to sigma‐receptors was proved by silencing the expression of σ‐receptors using anti σ‐receptor siRNA; this resulted in an increase in cell viability indicating a reduction in σ‐receptor—HP‐C8 binding.

Another well‐validated and common cancer targeting strategy involves the FR which is over‐expressed in many different cancer cells. Two FR targeting cationic lipids FA8 and FA12 with saturated, unbranched C8 and C12 alkyl chains, respectively were designed with folic acid as the head group (Figure A1Q).^[^ ^82^ ^]^ FA8 (Figure 8B) is a cationic lipid designed to target the FR by conjugating folic acid to a quaternary ammonium cationic lipid bearing twin C8 carbon chains. The lipid successfully delivered NME2 (a drug like molecule) to melanoma cells known to express moderate levels of FRs. The concept was proven in vivo using a melanoma tumor model; significant tumor regression was observed.

##### Brain‐Targeting Cationic Lipids

Drugs can be selectively delivered to the brain by actively targeting the receptors expressed on the cell surface of brain tissues. Nipecotic acid is a competitive inhibitor of the GABA transporter which is one such receptor.^[^ ^107^ ^]^ The cationic lipid hybrid NACA (nipecotic acid‐derived Cationic Amphiphile) (Figure 8C) was synthesized by incorporating nipecotic acid in the head group region of a quaternary ammonium cationic lipid bearing twin saturated, straight hexadecyl C16 hydrocarbon chains.^[^ ^108^ ^]^ Receptor selectivity of the NACA lipid was tested by pre‐treating neuroblastoma IMR‐32 cells with a GABA antibody which binds to the GABA transporter. Cellular uptake of NACA liposomes encapsulating labelled siRNA was lower in GABA antibody cells which prevent binding of the NACA lipid to the receptor. In vivo biodistribution studies in neuroblastoma (IMR‐32) bearing athymic nude mice using fluorescently labelled siRNA (FAM‐siRNA) encapsulated within NACA liposomes indicated that the liposomes primarily accumulated in the brain tumor tissues, with significantly lower accumulation detected in spleen, kidney, and lung tissues. A NACA liposomal formulation was also successfully used for combination therapy with co‐delivery of CDC20 siRNA and the chemotherapeutic paclitaxel in athymic nude mice bearing xenografted human neuroblastoma: cell death and tumor growth inhibition were successfully observed.

**Figure 17:**
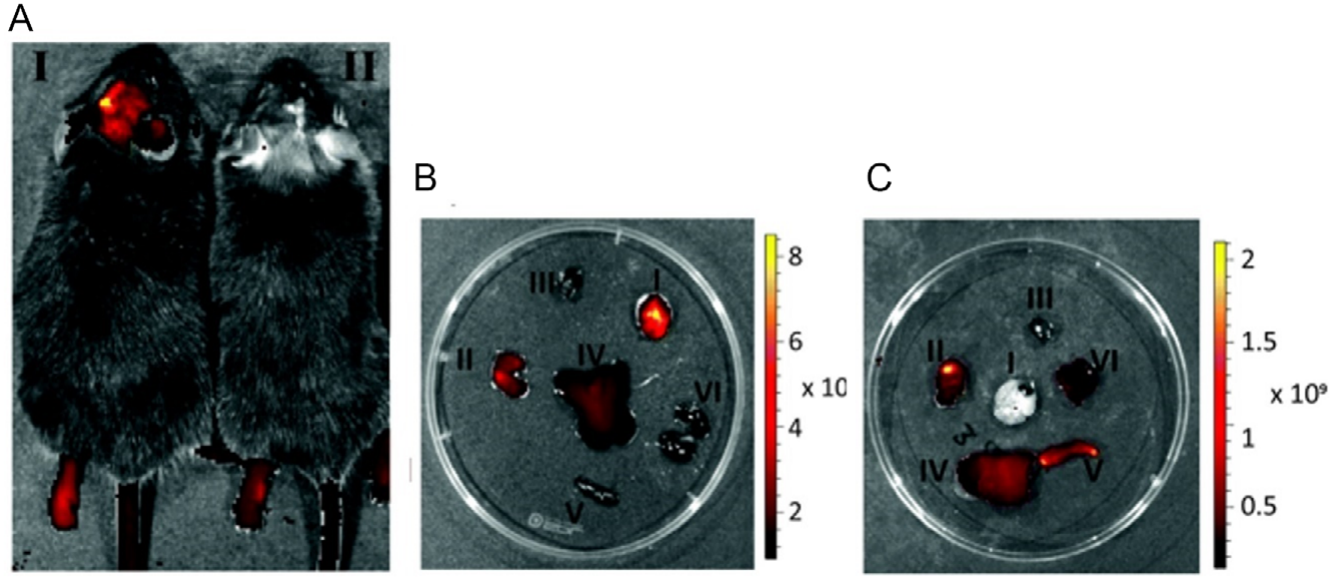
In vivo biodistribution studies in orthotopic glioblastoma C57BL/6 mice models. Ten post glioblastoma implantation mice were injected intravenously with both the fluorescently labelled nanoparticle of 16‐BACL‐ and NT‐lipid, A) 24 h later the intensity of the NIR‐dye was assessed using a non‐invasive imager I) Mice treated with the NIR‐loaded lipid nanoparticle of 16‐BACL and II) mice treated with the NIR‐loaded non‐targeting control lipid nanoparticle. B) ex vivo images of different organs isolated from mice treated with the NIR‐dye loaded lipid nanoparticle of 16‐BACL and C) the NIR‐dye loaded non‐targeting lipid nanoparticle were recorded. In (B) and (C), I) brain; II) lungs; III) heart; IV) liver; V) spleen; and VI) kidneys. Reproduced with permission.^[^ ^109^ ^]^ Copyright 2020, The Royal Society of Chemistry.

Three brain targeting cationic lipid hybrid analogues^[^ ^109^ ^]^ were developed based on *β*‐amphetamine, a lipophilic psychostimulant with demonstrated ability to penetrate the BBB. 14‐BACL (*β*‐amphetaminylated cationic lipid), 16‐ BACL, 18‐ BACL (Figure A1R) bear twin saturated, unbranched hydrocarbon chains of length C14, C16 and C18, respectively. Biodistribution studies in glioblastoma mice models (**Figure** **17**) showed that all three lipid nanoparticles accumulated in the brain; the C16 carbon chain analogue 16‐BACL (Figure 8D) exhibited maximum accumulation indicating the role of chain length in BBB penetration. Lipid nanoparticles based on 16‐BACL successfully encapsulated paclitaxel and PDL1‐siRNA and co‐delivered them both in vitro and in vivo improving the overall survivability of the orthotopic glioblastoma mice models compared to non‐targeting counterparts.

##### Liver‐Targeting Cationic Lipids

Asialoglycoprotein receptors are generally expressed on hepatocytes (liver cells) and several cancer cells. These receptors have affinity for sugar molecules, hence conjugating cationic lipids to sugar head groups imparts targeting ability to the cationic lipid hybrid. In this line cationic glycolipids bearing d‐galactose head (cyclic head (lipids 1–5) (Figure A1S(i)) and open head (lipids 6–10) (Figure A1S(ii)), twin saturated, unbranched C16 hydrocarbon chain and spacer units varying in length from 1 to 9 were synthesised.^[^ ^110^ ^]^ Two of these lipids effectively targeted genes to human hepatocarcinoma (HepG2) cells and primary hepatocytes via interactions with the asialoglycoprotein receptors: lipid 3 (Figure 8E), bearing a six carbon (C6) spacer among the cationic glycolipids with cyclic sugar head group and lipid 6 (Figure 8F), bearing a short, two carbon spacer (C2) among the cationic glycolipids with open sugar head group indicating the role of spacer in the interaction of sugar moiety with the asialoglycoprotein receptor. A decreased uptake of these glycolipids (3 and 6) by primary hepatocytes was observed when mice were pre‐treated with asialofetuin (which is a competitive ligand for asialoglycoprotein) confirming that the uptake is asialoglycoprotein mediated. Lipids 3 and 6 were both equally efficient in transfecting mouse livers in vivo suggesting that the chemical structure of the sugar (open chain or closed ring) did not impact the transfection efficiency.

##### Immune Cells‐Targeting Cationic Lipids

Dendritic cells are antigen‐presenting cells which process pathogenic antigens after their uptake from peripheral blood and tissues. The processed antigens are then presented to the T‐lymphocytes which mount an immune response to control the pathogens. Many proposed cancer vaccines rely on transfecting macrophages with DNA coding for the cancer antigens, thus triggering an immune response against the cancer cells. As dendritic cells harbour mannose receptors on their surface, mannose receptor targeting shikimoyl (lipid 1) (Figure 8G)‐, quinoyl (lipid 2) (Figure A1T(i))‐ and mannosyl (lipid 3) (Figure A1T(ii))‐ cationic lipids with twin C16 straight, saturated hydrophobic tails separated by a lysine spacer (guanidylated) were synthesised to encapsulate and deliver a DNA‐based vaccine (p‐CMV‐MART1) encoding the melanoma antigen to macrophages.^[^ ^111^ ^]^ In vivo studies showed that systemic administration of the lipoplexes induced a long‐lasting antimelanoma response with Lipid 1 bearing the shikimoyl head group (Figure 8G) being the most efficient.

##### Lung‐Targeting Cationic Lipids

A pyridinium‐based cationic lipid 1‐(2,3‐dioleoyloxypropyl)‐2,4,6‐trimethylpyridinium lipid, 2OC (Section 4.1; Figure 3A(iii)), demonstrated improved transfection in lung cancer cell lines,^[^ ^73^ ^]^ relative to the commercially available DOTAP. This was attributed to the presence of polyamine transporters specific for pyridinium molecules in lung cells. The results agreed with an associated in vivo assay in nude mice.

**Figure 18:**
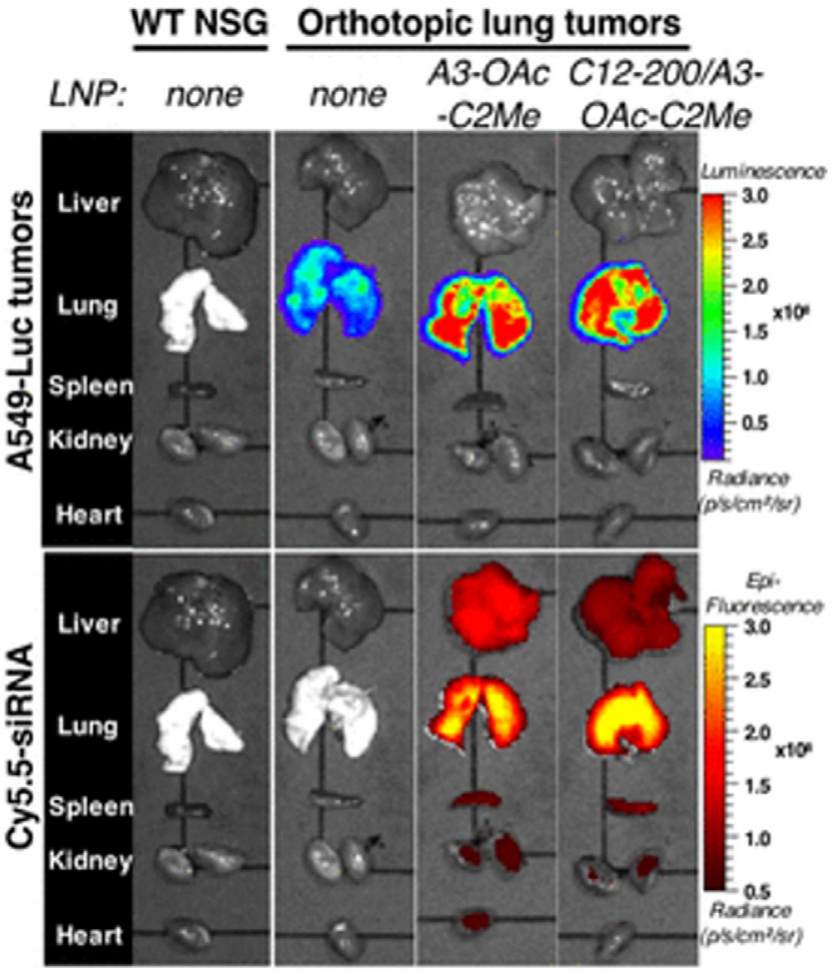
CSAL LNPs lung targeting A549‐Lucorthotopic tumors in mouse lungs. Ex vivo organ imaging shows that CSAL LNPs enable delivery to cancerous lungs. Top‐luminescence visualization of tumors by BLI, Bottom fluorescence imaging using cy5.5 siRNA showed strong lung accumulation. Reproduced with permission.^[^ ^112^ ^]^ Copyright 2018, American Chemical Society.

In another study, the cationic lipid, cationic quaternary ammonium sulfonamide amino lipids (CSAL) (Figure 8H) demonstrated a tendency to accumulate in the lungs (**Figure** **18**). This selectivity was demonstrated by including CSALs as excipients in liver targeting C12‐200 lipid nanoparticles (LNPs), a shift in the biodistribution of the LNPs toward the lungs was observed.^[^ ^112^ ^]^

#### 4.3.4 Therapeutic Lipids

##### Anticancer‐Cationic Lipid Hybrids

The sex steroid hormones estrogen and progesterone are known to play a significant role in the development and growth of breast cancers by binding to the endogenous estrogen and progesterone nuclear receptors, respectively and activating a signalling cascade downstream. Among the library of estradiol cationic lipid hybrids with unbranched, saturated carbon chains with length ranging from C6 to C14 (Figure A1U), estradiol conjugated to C8 and C10 carbon chains, ESC8^[^ ^71^ ^]^ and ESC10^[^ ^121^ ^]^ (Figure 9A) exhibited anti‐breast cancer activity regardless of the estrogen receptor (ER) expression status being active both on ER +ve (Estrogen receptor positive) and ER−ve (Estrogen receptor negative) breast cancers. In contrast, selective estrogen receptor modulators (SERMs), commercial anti‐breast cancer chemotherapeutics, kill only ER +ve breast cancers. In addition, the estradiol cationic lipid hybrids had lower IC_50_ values than the commercial non‐estrogen SERMs 4‐hydroxytamoxifen (4OH‐Tam) and 2‐methoxyestradiol (2OMe‐ES). The 10‐carbon chain analogue, ES C10, was found to be 4‐12‐fold more active than 8‐carbon analogue ESC8. However, ESC‐8 demonstrated good oral bioavailability when formulated into solid lipid nanoparticles (ESC‐8 SLNs) when tested in Sprague‐Dawley rats.

In a similar manner, progesterone, a steroid sex hormone, was conjugated to twin saturated, straight C10 chain cationic lipids resulting in PR 10, another sex steroid‐cationic lipid hybrid (Figure 9B).^[^ ^117^ ^]^ The molecule was observed to be active against progesterone receptor‐positive (PgR +ve) (T47D) and progesterone receptor—negative(PgR −ve) (B16F10) cell lines in both in vitro and in vivo studies. Tumors are associated with angiogenesis, a condition where new blood vessels are formed from pre‐existing ones; molecule PR 10 was also found to have significant antiangiogenic activity inducing apoptosis in the tumor and associated vasculature.

**Figure 19:**
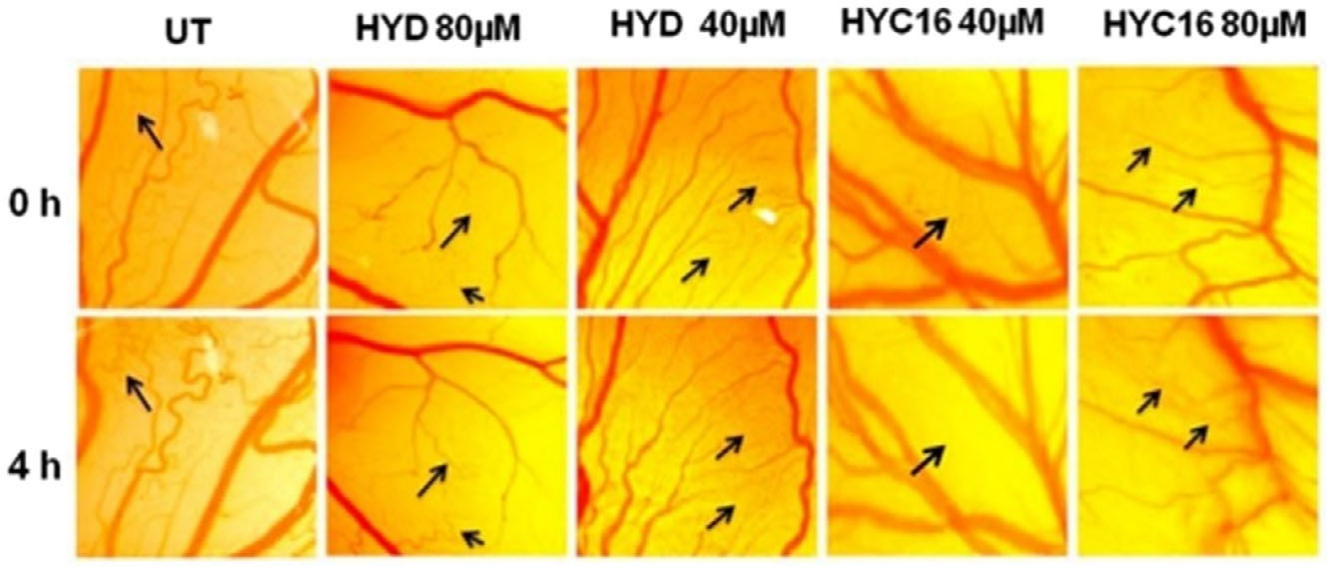
Anti‐angiogenesis effect of HYC16 ‐ Chick embryo angiogenesis (CEA) assay. Chick embryo model treated with HYC16, HYD (at 40 and 80 μM) or kept untreated. Reproduced with permission.^[^ ^116^ ^]^ Copyright 2015, Elsevier Masson SAS.

Similarly, hydrocortisone, which is involved in the pathophysiology of various cancers, was conjugated to a quaternary ammonium cationic lipid of saturated, unbranched hydrocarbon chains ranging in length from C8–C16. Analogue with twin C16 hydrocarbon chains HYC16 (Figure 9C)^[^ ^116^ ^]^ exhibited selective cytotoxicity toward lung, breast and melanoma cancer cells, with reduced toxicity toward normal cell lines. The full library of hydrocortisone cationic lipids synthesized is presented in the Figure A1V. The tumor shrinkage brought about by the HYC16 is reported to be due to its antiangiogenic activity (**Figure** **19**).

**Figure 20:**
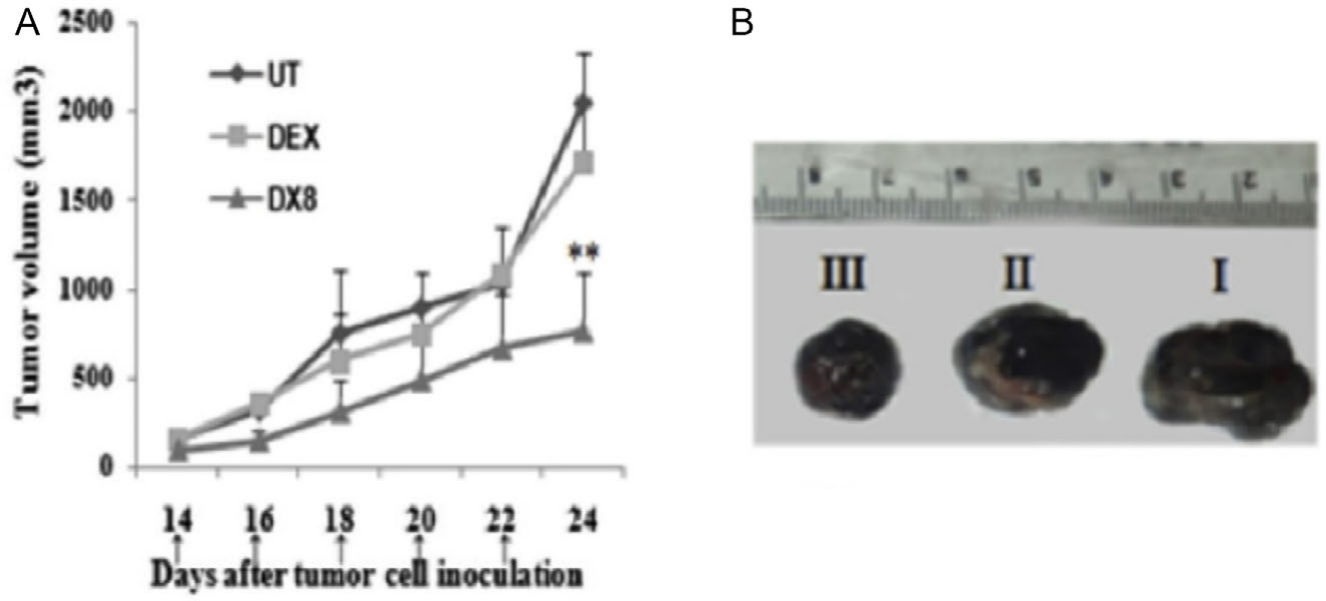
In vivo studies of DX8 in B16F10 cells implanted C57BL6/J mice models. A) Tumor regression curve after treatment with Dex (Dexamethasone), DX8 (cationic lipid), untreated (UT). Black arrows—days on which the mice are injected. DX8 induced significant tumor regression (*p* < 0.05) compared to Dex treatment. B) Images of tumors I) untreated (UT), II) Dex‐treated, III) DX8‐treated. Reproduced with permission.^[^ ^118^ ^]^ Copyright 2014, Elsevier Masson SAS.

Dexamethasone, used as a palliative medicine in cancer treatment to reduce the antiproliferation and inflammatory syndromes associated with chemotherapy treatment, was imparted with potent and selective anticancer activity when covalently conjugated to a twin, saturated straight carbon chain quaternary ammonium cationic lipid (Figure A1W).^[^ ^118^ ^]^ Among the library of DX cationic lipid hybrids with even numbered carbon chain lengths ranging from C2 to C16 carbons, DX8 (Figure 9D), the 8 carbon chain analogue, exhibited cancer selective anti‐proliferative activity in lung, breast, and melanoma cancer cell lines. In vivo studies in a melanoma mice model demonstrated significant tumor regression in the group treated with DX8 compared to untreated and Dexamethasone treated groups (**Figure** **20**). DX10, a C10‐carbon chain analogue (Figure A1W), in combination with WP (a commercial STAT3 inhibitor) synergistically induced tumor regression in a melanoma mice model via apoptosis in both the tumor mass and the blood vessels associated with.^[^ ^140^ ^]^

When haloperidol, a neuropsychotic drug which exhibits poor anticancer activity by itself, was conjugated to a quaternary ammonium cationic lipid with saturated, unbranched twin alkyl chains its anticancer activity was potentiated. HP‐C8, the haloperidol analogue bearing twin C8‐carbon chains (Figure 8A; Section 4.2.4) in the solution form exhibited significant sigma receptor mediated anticancer activity in cancer cell lines compared to analogues with C4, C12, and C16 carbon chain lengths.^[^ ^105^ ^]^ HP‐C8 was also observed to control tumor aggressiveness in a mice melanoma model and exhibited potent antiangiogenic activity. Similarly, the anticancer activity of emodin (1,3,8‐trihydroxy‐6‐methyl‐9,10‐anthraquinone)—which is known to exert weak antitumor activity—was augmented when conjugated to quaternary ammonium cationic carbon chains of varying length (Figure A1X).^[^ ^122^, ^123^ ^]^

Stilbenes are anticancer agents whose activity is limited by their poor pharmacokinetic properties and non‐specific action. In this case lipidation was observed to improve the pharmacokinetic properties of the anticancer agents. Chemical modifications, including protection of the hydroxyl groups and conjugation of the molecule to twin saturated, unbranched chain cationic lipids has been shown to improve the activity of stilbenes (Figure A1Y). Among analogues with C8, C10, and C16 carbon chain lengths HMSC16 (Figure 9E), analogue with twin C16 carbon chain and all methoxy groups was observed to be most potent and cancer selective compared to the other novel stilbene analogues and the commercially available stilbene‐based molecules tamoxifen and resveratrol.^[^ ^120^ ^]^ No significant differences were observed in the IC_50_ of the neat lipid and the self‐aggregate when tested in HeLa cell lines.

Cordiarimide, a natural product with weak anticancer activity, was conjugated to twin saturated unbranched alkyl chains with chain lengths ranging from C2 to C14 carbons (Figure A1Z(i)). NP C12, the cationic lipid hybrid with twin C12 chains (Figure 9G) demonstrated not only improved anticancer activity but was also imparted with the ability to inhibit human DNA ligase I, a DNA repair enzyme which is implicated in the development of resistance to conventional chemotherapy.^[^ ^70^ ^]^

**Figure 21:**
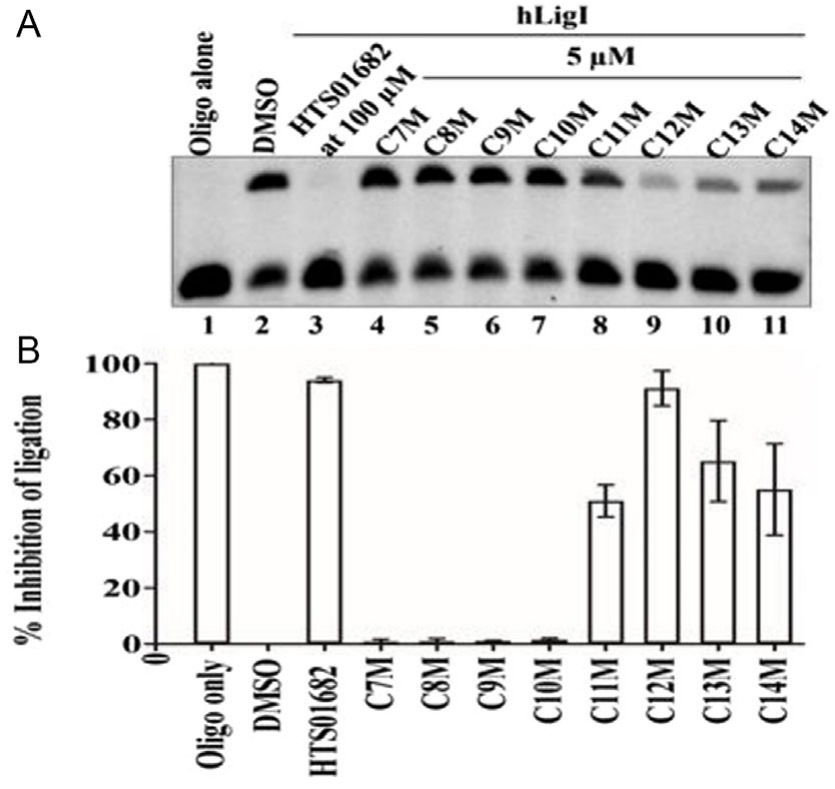
A) Agarose gel image showing the hLig I inhibition activity of the cationic lipids C7M−C14M with varying chain lengths (Lanes 4–10), positive control HTS 01682 (Lane 3), negative control without any inhibitor (Lane 4) and Cy‐3 labelled DNA as substrate control (Lane 1). B) Graph showing the quantified % inhibition of hLigI activity of C7M−C14M compounds. Reproduced with permission.^[^ ^115^ ^]^ Copyright 2018, American Chemical Society.

Lipobenzamides were synthesized by conjugating benzamide, a small organic molecule with negligible biological activity, to saturated, straight twin chain quaternary ammonium cationic lipids of chain length ranging from C2–C18 carbons (Figure A1Z(ii)). C10M an analogue with twin C10 carbon chains (Figure 9F(top)) was reported to inhibit cancer cell proliferation selectively, and was able to self‐assemble, encapsulate and deliver anticancer siRNA to prostate cancer DU‐145 cell lines.^[^ ^114^ ^]^ The analogue with twelve carbon chains, C12M (Figure 9F(bottom)), was reported to significantly inhibit human DNA ligase 1 (**Figure** **21**), an enzyme overexpressed in cancer cells which is also involved in the development of chemotherapy drug resistance. C12M successfully formed liposomes in combination with cholesterol and delivered anti‐survivin siRNA resulting in an enhanced anticancer response both in vitro and in vivo.^[^ ^115^ ^]^

Self‐assembly of these lipid drug conjugates into nanocarriers such as liposomes allows co‐delivery of either a chemotherapeutic or a biotherapeutic like nucleic acids (siRNA or shRNA) or both for cancer therapy, typically demonstrating improved efficacy relative to conventional monotherapy.^[^ ^141^ ^]^ HMSC16 (Figure 9E), a stilbene cationic lipid hybrid, was reported to self‐aggregate (the morphology of the aggregated particles was not specified) and was shown to encapsulate hydrophobic drugs such as Withaferin (a steroidal lactone having anticancer properties); the combination was reported to have additive cytotoxic effect in HeLa cells. Thus, HMSC16 not only acts as a drug but also as a drug delivery agent. When formulated with equimolar cholesterol, C12M (Figure 9F (bottom)) successfully transfected survivin siRNA and showed significant tumor inhibition in vivo. HYC16 (Figure 9C), a hydrocortisone cationic lipid hybrid, was also observed to self‐assemble into vesicles^[^ ^119^ ^]^ which was confirmed by TEM. HYC16 efficiently co‐encapsulated both docetaxel and p53 plasmid DNA and delivered successfully to colorectal cancer cells both in vitro and in vivo.

##### Anti‐Microbial Lipid Hybrids

Grouping several cationic head groups into a single molecule was shown to enhance the antibacterial activity. Biodegradable carbohydrates including starch, cyclodextrin, and monosaccharide glycosides were used as scaffolding to conjugate the doubly positively charged DABCO bearing a single chain (Figure 10A).^[^ ^130^ ^]^ The synthesized molecules were tested against Gram‐positive bacteria (*Staphylococcus aureus*), Gram‐negative bacteria, (*Pseudomonas aeruginosa*), and fungi (*Candida albicans* and *Aspergillus* *niger*). It was shown that single molecules of starch (polysaccharide) accommodated a higher number of positively charged DABCO units compared to the cyclodextrin or monosaccharide glycoside. It was also observed that cationic lipid units attached to the starch were 5 × 10^4^ times more antimicrobial than the cyclodextrin or monosaccharide analogues.

The antimicrobial activity of sophorolipids, which are amphiphilic molecules consisting of a hydrophilic sophorose head group and a hydrophobic oleic acid tail, was enhanced by conjugation to a quaternary ammonium lipid. The synthetic sophorolipids were evaluated for their antimicrobial properties against both Gram‐negative strains (*E. coli*, *K. pneumoniae*, *P. aeruginosa* PAO1) and Gram‐positive strains (*S. aureus*, *B. subtilis*, *Enterococcus faecium*, *Streptococcus pneumoniae*). The antimicrobial efficacy depended on several molecular features including the total length of the sophorolipid tail, the length of the lipid chain attached to the nitrogen, and the position of the quaternary nitrogen from the sophorose group. Among the peracetylated lipids with 8‐carbon spacer between the sugar moiety and quaternary ammonium group, unbranched saturated C18 carbon chain analogues 11 h (R_3_ = CH_3_) and 11i (R_3_ = C_4_H_9_) (Figure 10B) were observed to significantly inhibit *S. aureus* and *B. subtilis* while deprotected analogues 3h (R_3_ = CH_3_) and 3i (R_3_ = C_4_H_9_) (Figure 10C) were active only against Gram‐positive strains.^[^ ^131^ ^]^ All four synthetic sophorolipids 11h, 11i, 3h, and 3i were found to be more active than the antibiotic gentamicin sulfate against the four Gram‐positive strains. The peracetylated, saturated, straight C12 carbon chain analogue with an 11‐carbon spacer between the sugar moiety and quaternary ammonium group, 11d (Figure 10D), was observed to perform better compared to 3h and 3i.^[^ ^132^ ^]^ Microscopic analysis revealed that lysis of the cells occurred at the active concentrations. Sophorolipids were observed to form micelles and their cytotoxicity was found to be due to their ability to form micelles.

Two series of cationic lipoxazoles 5(a‐h) (without CF_3_ substitution), 6(a‐h) (with CF_3_ substitution) (Figure A1,AA), were developed and tested for antifungal activity against *C. albicans.* ^[^ ^133^ ^]^ It was observed that the hydrocarbon chain length affects the antifungal activity; analogues bearing saturated, straight C7 and C8 carbon chains from both series, 5c, 6a, 6b, 6c were reported to exhibit concentration‐dependent growth inhibition of *C. albicans*. Analogue 5c with a C8 chain (Figure 10E) was reported to be the most effective against both sensitive and resistant clinical isolates and non‐albicans strains at comparably low concentrations at which the compound was found to be non‐toxic to mammalian cells and erythrocytes. At higher concentrations 5c inhibited surface adhesion of *C.* albicans which is a crucial step in the biofilm formation. Antifungal activity was not due to the lysis but due to the membrane destabilization which was confirmed by scanning electron microscopy (SEM) imaging studies.

Lipobenzamide molecules, C2M‐C18M, were also tested for their antifungal activity against the fungal strain *C. albicans* along with anticancer screening (Figure A1Z(ii)).^[^ ^134^ ^]^ It was observed that compounds having unbranched, saturated medium alkyl chain lengths with odd numbers of carbons, that is, C9 carbon chain analogue C9M (Figure A1Z(ii) *n* = 7) and C11 carbon chain analogue C11M (Figure A1Z(ii) *n* = 9), were found to be most active against *C. albicans* (ATCC 90028 strain). C9M inhibited the growth of both fluconazole‐sensitive and resistant clinical isolates of *C. albicans* equally and was also active against non‐albicans strains. While the commercial antifungal fluconazole failed to inhibit the *C. albicans* biofilm formation, C9M successfully inhibited the biofilm at 12.5 μg mL^−1^. C9M was observed to be both biocompatible with mammalian cells (cancerous and non‐cancerous) and red blood cells at a concentration (10 μg mL^−1^) at which it is active toward *Candida species*. SEM imaging studies indicate that the antifungal activity of the molecule was due to the membrane destabilizing effect.

Thus, unlike earlier generations of cationic surfactants such as DODAB, some newer antimicrobial surfactants are selective toward bacterial cells while remaining non‐toxic to mammalian cells at the doses effective for antimicrobial activity. This can be achieved by conjugating targeting ligands to the quaternary ammonium cationic lipid. However, similar to anticancer cationic lipid hybrids only a specific combination of chain lengths works for different head groups. For example, in the case of sophorolipids analogues with saturated, straight C18 and C11 carbon chain length exhibited better antimicrobial activity. For the lipoxazole head group, a saturated moderate carbon chain length C8 resulted in an active analogue. In the case of the lipobenzamide head group, the saturated odd carbon chain lengths C9 and C11 were observed to be active.

##### Antioxidant Lipid Hybrids

One issue with cationic lipids is that they are known to induce intracellular ROS (Reactive Oxygen Species) which can trigger the necroptosis cascade.^[^ ^67^ ^]^ Therefore, to develop safer drug delivery vectors, researchers have developed cationic lipid hybrids having intrinsic antioxidant properties to quench the ROS generated. A series of ionizable (tertiary ammonium) cationic lipids TOC‐As^[^ ^142^ ^]^ and zwitterionic lipid LOPC,^[^ ^143^ ^]^ bearing antioxidant properties were developed. Similarly, a series of permanently charged lipophilic morpholinium quaternary ammonium salt analogues bearing an antioxidant phenol moiety were developed (Figure 11).^[^ ^135^ ^]^

Uptake of these lipids into model erythrocyte membranes at sublytic concentrations was reported. Fluorescence experiments on erythrocytes labelled with the fluorescent probes DPH and TMA–DPH indicated that an increase in lipophilicity of the analogues resulted in an increase in membrane fluidity consistent with longer alkyl chain analogues being well incorporated into the erythrocyte membrane. Spectrophotometric measurements of the concentrations of the malone dialdehyde generated as result of the lipid peroxidation, revealed that the antioxidant activity of the morpholinium analogues with the long alkyl chains was observed to be high, with PPMA‐15 (Figure 11) bearing unbranched, saturated C15 carbon chain length exhibiting four times better antioxidant property compared to the PPMA‐7 (Figure 11) bearing saturated C7 carbon chain, which was attributed to increased uptake of these longer chains into the membrane resulting in improved protection. While hemolytic studies with pig erythrocytes indicated that longer alkyl chains were associated with increased hemolytic activity, the hemolysis was generally observed to happen at a higher dose than that required for antioxidant activity.

## 5 Conclusions and Future Perspectives

This review summarises a very wide range of cationic lipid hybrids with potential applications in transfection, targeting and as therapeutic lipids or bioactive excipients. In almost all cases, the specific biological response required is elicited via modifications to the headgroup region of the cationic lipid. Effective transfection efficiency requires the headgroup to be positively charged, such as with gemini cationic lipids which contain two units of cationic charge per molecule. This facilitates encapsulation of the negatively charged RNA and enhances the interaction with typically anionic cell membranes. Inclusion of polar groups, such as hydroxy or amine groups, was also shown to increase transfection efficiency; it was suggested that this may reflect increased hydrogen bonding between the cationic lipid and the nucleic acid molecule which can enhance uptake. However, the high toxicity associated with this positive headgroup charge may be tempered by additional modifications to the headgroup such as the inclusion of heterocyclic rings such as imidazolium, pyridinium, or guanidinium group which delocalize the charge.

Effective targeting of a cationic lipid in vitro or in vivo also focussed on headgroup modifications. In the vast majority of cases, including for targeting cancer cells in the brain, lungs, and liver, known receptor–ligand interactions are invariably utilized with the headgroup modified to include the ligand.

Finally, the efficacy of therapeutic cationic lipids, whether these are anticancer, antimicrobial, or antioxidant generally rely mainly on modifications to the headgroup region of the cationic lipid. For anticancer lipids, this generally involves conjugation of a known pharmacophore with a range of studies demonstrating enhancement of the efficacy of known chemotherapeutics via conjugation to a cationic lipid. It is thought that lipid conjugation may also avoid or slow down the development of drug resistance in some tumours by masking the drug itself. In some cases, drugs which display either no or weak anticancer activity were rendered chemotherapeutic via the inclusion of one or more alkyl chains. The efficacy of antimicrobial cationic lipid hybrids generally relies on the positive charge of a quaternary ammonium group in the headgroup region which increases disruption of bacterial and fungal surfaces.

However, although the biological effect is generally governed by the headgroup architecture, modifications to the hydrocarbon chain and/or a spacer have been shown to mediate and enhance the desired effect in vitro and in vivo and are critically important to the design of effective cationic lipid hybrids. Some very broad trends are observed with, for example, medium chain length lipids in the range C8–C12 generally observed to result in higher biological efficacy for a range of different cationic lipids. We note several exceptions to this trend, particularly for longer‐chain (C18) unsaturated lipids which may be more likely to partition into biological cell membranes. As many studies only investigate a small number of different chain lengths and architectures, at this stage it is very difficult to directly compare between different research studies. Another issue relates to the fact that many studies investigate the effect of the lipid headgroup separately to that of the lipid hydrocarbon chain architecture. However, changes to either molecular feature will change the average molecular “shape” as quantified by the critical packing parameter of these lipids which is known to impact the interactions of these lipids with cells modifying their biological efficacy. Future studies should attempt to estimate the CPP of the novel synthesised lipids to fully elucidate the effect on the desired end‐behaviour.

Advances in high‐throughput synthesis and screening of cationic lipids will allow for the systematic investigation of a wider range of different hydrocarbon chain lengths, as well as features such as unsaturation and branching. Such studies must be combined with robust machine learning computational studies to determine which molecular features of the headgroup, linker, and hydrocarbon chain region are associated with improved features such as high transfection efficacy, low toxicity, and good serum compatibility and to more effectively select appropriate lipids to advance to animal studies and pre‐clinical testing.

Finally, the impact of the self‐assembly behaviour of these novel cationic lipids on their biological efficacy remains significantly under‐explored. Most studies don't comment on any potential self‐assembly of the cationic lipid into micelles, liposomes, or other more complex lipid nanoparticles such as cubosomes or hexosomes. As the self‐assembly structure is known to impact the interactions and efficacy of lipid‐based moieties in vitro and in vivo this must be more thoroughly understood if the full potential of novel cationic lipid hybrids for therapeutic applications in vivo is to be realized.

## Conflict of Interest

The authors declare no conflict of interest.
